# Supplementary material for: Intermarriage and COVID-19 mortality among immigrants. A population-based cohort study from Sweden
Source: BMJ Open. 2021 Aug 31;11(9):e048952. doi: 10.1136/bmjopen-2021-048952 (PMC8413476; doi:10.1136/bmjopen-2021-048952)
Supplement: Supplementary data [file bmjopen-2021-048952supp001.pdf]

Supplementary table 1: Descriptive table displaying distributions of all covariables with and without missing in education and dwelling size.

	Study population (with missing education and dwelling size)	Study population (without missing education)	Study population (without missing dwelling size)
**Couple dyads**			
Native-native	75.31	75.86	75.25
Native-immigrant	6.18	6.21	6.19
Immigrant-native	6.09	5.99	6.09
Immigrant-immigrant	12.42	11.94	12.47
**Sex**			
Man	50.26	50.21	50.26
Woman	49.74	49.79	49.74
**Education**			
Primary	14.89	15.01	14.91
Secondary	42.59	42.95	42.58
Post-secondary	41.68	42.04	41.66
Missing	0.85		0.85
**Income HH disposable Inc (tertlies)**			
1	32.53	32.22	32.58
2	33.72	33.85	33.73
3	33.75	33.93	33.69
**Housing type**			
Multi-family dwelling	32.38	32.11	32.64
Single-family dwelling	67.18	67.47	66.91
Care home	0.44	0.42	0.45
**# of individuals in the househould**			
2	52.46	52.47	52.50
3	15.52	15.50	15.53
4	21.97	22.02	21.93
5+	10.05	10.01	10.04
**Sqm per person**			
0	8.21	8.08	8.28
-20	20.55	20.50	20.71
-30	23.04	23.04	23.23
-40	28.45	28.54	28.69
-60	18.94	19.03	19.10
Missing	0.81	0.81	
**Immigrants in DeSO**			
0	32.97	33.15	32.77
.1-	23.81	23.88	23.82
.15-	16.63	16.64	16.70
.2-	14.03	14.01	14.09
.3-	8.81	8.72	8.86
.5-	3.73	3.61	3.76
**Region of residence**			
Stockholms län	21.11	21.01	21.13

Uppsala län	3.57	3.57	3.57
Södermanlands län	2.91	2.92	2.90
Östergötlands län	4.64	4.65	4.65
Jönköpings län	3.73	3.74	3.73
Kronobergs län	2.05	2.04	2.05
Kalmar län	2.60	2.60	2.60
Gotlands län	0.59	0.60	0.59
Blekinge län	1.68	1.68	1.67
Skåne län	13.24	13.20	13.24
Hallands län	3.59	3.60	3.59
Västra Götalands län	16.71	16.73	16.75
Värmlands län	2.89	2.88	2.89
Örebro län	2.95	2.96	2.95
Västmanlands län	2.74	2.74	2.75
Dalarnas län	2.95	2.96	2.93
Gävleborgs län	2.89	2.91	2.90
Västernorrlands län	2.56	2.57	2.56
Jämtlands län	1.29	1.30	1.28
Västerbottens län	2.75	2.76	2.73
Norrbottens län	2.56	2.57	2.54

Supplementary table 2: Regression results for the risk of dying from COVID-19, other causes of death during the pandemic, and all-cause mortality in the year prior to the pandemic by couple dyad

	Model 1: Figure 2A						Model 2: Figure 2B					
	COVID- 19		Other COD Mar 5, 2020- Feb 23, 2021		All COD Mar 5, 2019 – Feb 23, 2020		COVID- 19		Other COD Mar 5, 2020- Feb 23, 2021		All COD Mar 5, 2019 – Feb 23, 2020	
	HR	95% CI	HR	95% CI	HR	95% CI	HR	95% CI	HR	95% CI	HR	95% CI
**Partnership type**												
Native-native	1.00	1.00	1.00	1.00	1.00	1.00	1.00	1.00	1.00	1.00	1.00	1.00
Native-immigrant	1.40	(1.25, 1.58)	1.07	(1.02, 1.13)	1.10	(1.05, 1.15)	1.15	(1.02, 1.29)	1.03	(0.98, 1.08)	1.05	(1.00, 1.10)
Immigrant-native	1.50	(1.33, 1.69)	1.00	(0.95, 1.06)	1.11	(1.05, 1.16)	1.24	(1.10, 1.40)	0.98	(0.93, 1.03)	1.07	(1.02, 1.12)
Immigrant-immigrant	2.47	(2.27, 2.69)	1.08	(1.04, 1.13)	1.04	(1.00, 1.09)	1.43	(1.29, 1.58)	0.90	(0.85, 0.94)	0.87	(0.83, 0.91)
Years under risk	3,759,610		3,759,610		3,764,370		3,759,610		3,759,610		3,764,370	
N events	4564		30,374		31,653		4564		30,374		31,653	
N	3,963,356		3,963,356		3,966,345		3,963,356		3,963,356		3,966,345	

Model 1 includes adjustments for sex; Model 2 includes adjustments for sex, household income, education, housing type, number of individuals in the household, m^2/person in the household (crowdedness), share of immigrants in the neighborhood of residence, and region of residence

Supplementary table 3: Regression results for the risk of dying from COVID-19, other causes of death during the pandemic, and all-cause mortality in the year prior to the pandemic by couple dyad disaggregated by LMIC and HIC immigrants.

	Model 3: Figure 3A						Model 4: Figure 3B					
	COVID- 19		Other COD Mar 5, 2020- Feb 23, 2021		All COD Mar 5, 2019 – Feb 23, 2020		COVID- 19		Other COD Mar 5, 2020- Feb 23, 2021		All COD Mar 5, 2019 – Feb 23, 2020	
	HR	95% CI	HR	95% CI	HR	95% CI	HR	95% CI	HR	95% CI	HR	95% CI
**Partnership type**												
Native-native	1.00	1.00	1.00	1.00	1.00	1.00	1.00	1.00	1.00	1.00	1.00	1.00
Native-immigrant	1.41	(1.25, 1.58)	1.07	(1.02, 1.13)	1.10	(1.05, 1.15)	1.16	(1.03, 1.30)	1.03	(0.98, 1.08)	1.04	(0.99, 1.09)
HIC-native	1.44	(1.27, 1.64)	1.01	(0.96, 1.07)	1.13	(1.07, 1.19)	1.23	(1.08, 1.40)	0.99	(0.94, 1.05)	1.11	(1.05, 1.16)
HIC-immigrant	1.54	(1.34, 1.77)	1.13	(1.07, 1.20)	1.09	(1.03, 1.16)	1.11	(0.97, 1.28)	1.01	(0.95, 1.07)	0.97	(0.91, 1.02)
LMIC-native	1.91	(1.42, 2.57)	0.95	(0.83, 1.09)	0.96	(0.84, 1.09)	1.35	(1.00, 1.82)	0.85	(0.74, 0.98)	0.84	(0.74, 0.97)
LMIC-immigrant	3.60	(3.25, 3.99)	1.03	(0.97, 1.10)	0.99	(0.93, 1.06)	1.84	(1.62, 2.09)	0.77	(0.72, 0.83)	0.75	(0.70, 0.81)
Years under risk	3,759,610		3,759,610		3,764,370		3,759,610		3,759,610		3,764,370	
N events	4564		30,374		31,653		4564		30,374		31,653	
N	3,963,356		3,963,356		3,966,345		3,963,356		3,963,356		3,966,345	

Model 3 includes adjustments for sex; Model 4 includes adjustments for sex, household income, education, housing type, number of individuals in the household, m^2/person in the household (crowdedness), share of immigrants in the neighborhood of residence, and region of residence

Supplementary figure 1: Hazard Ratios for the risk of dying from COVID-19 by couple dyad, including estimates from sensitivity analyses using multiple imputations to adjust for missing education and only complete cases

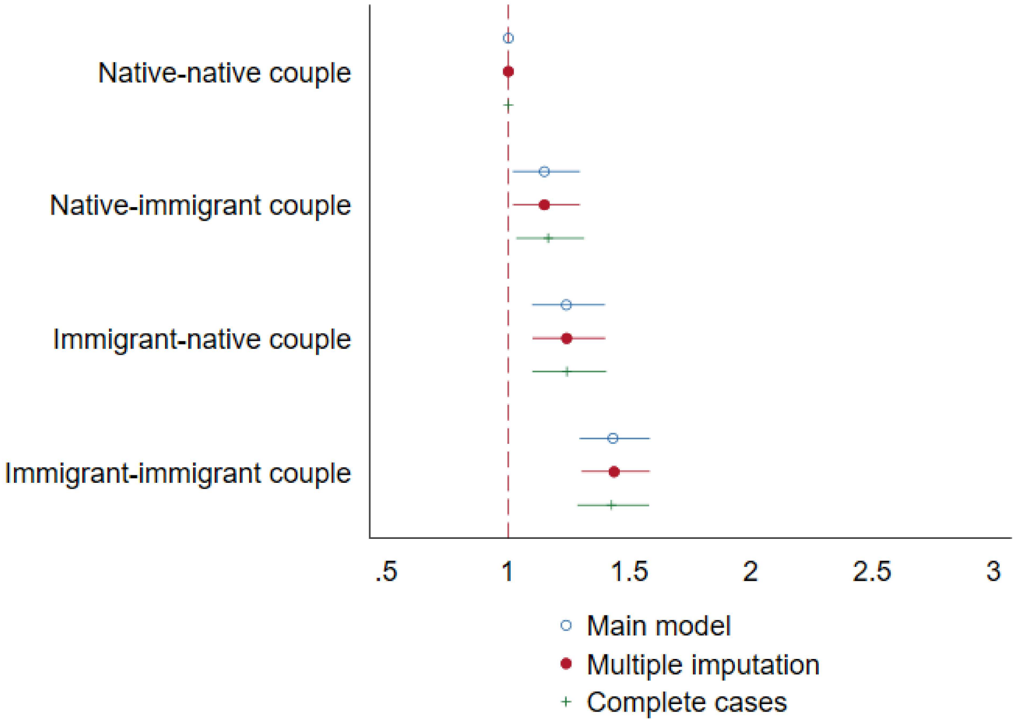

Supplementary figure 2: Hazard Ratios for the risk of dying from COVID-19, other causes of death during the pandemic, disaggregating native-immigrant couples by partner origin. Model includes full adjustment (reference group: native-native couples) (N=3,229,772)

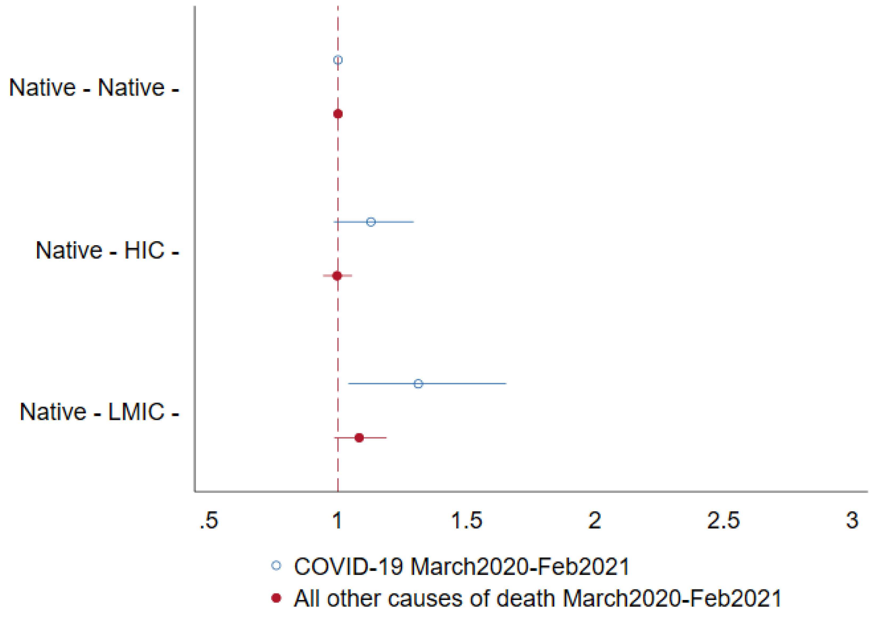

Supplementary figure 3: Hazard Ratios for the risk of dying from COVID-19 by couple dyads excluding second generation immigrants. Model includes full adjustment (reference group: native-native couples) (N= 3,593,764)

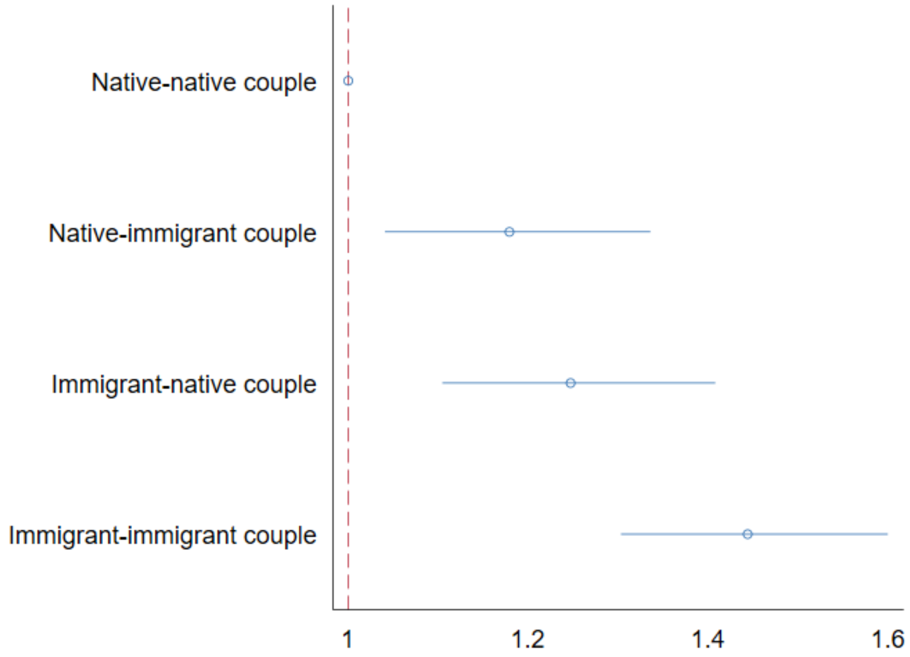

Supplementary figure 4: Hazard Ratios for (A) adjusted for age and sex only and (B) further adjusted associations between immigrant-native couple type (reference group: native-native couples), COVID-19 and all-cause mortality in 2020 in Stockholm, Sweden.

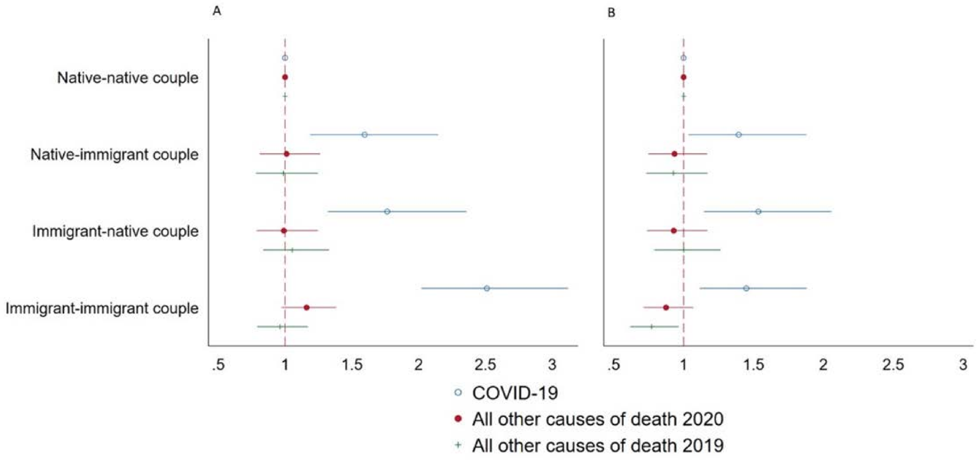

Note: This figure is based on data on all COVID-19 deaths during the period from March 12, 2020 to May 7, 2020 (149,622 person-years of observation, and 1,587 deaths).
