# Supplementary material for: Intermarriage and COVID-19 mortality among immigrants. A population-based cohort study from Sweden
Source: BMJ Open. 2021 Aug 31;11(9):e048952. doi: 10.1136/bmjopen-2021-048952 (PMC8413476; doi:10.1136/bmjopen-2021-048952)
Supplement: Author's manuscript [file bmjopen-2021-048952.draft_revisions.pdf]

BMJ Open

BMJ Open is committed to open peer review. As part of this commitment we make the peer review history of every article we publish publicly available.

When an article is published we post the peer reviewers' comments and the authors' responses online. We also post the versions of the paper that were used during peer review. These are the versions that the peer review comments apply to.

The versions of the paper that follow are the versions that were submitted during the peer review process. They are not the versions of record or the final published versions. They should not be cited or distributed as the published version of this manuscript.

BMJ Open is an open access journal and the full, final, typeset and author-corrected version of record of the manuscript is available on our site with no access controls, subscription charges or pay-per-view fees (http://bmjopen.bmj.com).

If you have any questions on BMJ Open's open peer review process please email info.bmjopen@bmj.com

BMJ Open

Lack of acculturation does not explain excess COVID-19 mortality among immigrants. A population-based cohort study

Journal:	*BMJ Open*
Manuscript ID	bmjopen-2021-048952
Article Type:	Original research
Date Submitted by the Author:	19-Jan-2021

Complete List of Authors:	Aradhya, Siddartha ; Stockholms Universitet, Stockholm University Demography Unit<br>Branden, Maria; Linköping University, The Institute for Analytical Sociology (IAS); Stockholms Universitet, Stockholm University Demography Unit<br>Drefahl, Sven; Stockholm University, Sociology<br>Obucina, Ognjen; Institut national d'études démographiques<br>Andersson, Gunnar ; Stockholm University, Department of Sociology<br>Rostila, Mikael ; Stockholms Universitet, Department of Public Health Sciences<br>Mussino, Eleonora; Stockholms Universitet, Stockholm University Demography Unit<br>Juárez , Sol Pia; Stockholm University, Department of Public Health Sciences

Keywords:	COVID-19, PUBLIC HEALTH, Demography < TROPICAL MEDICINE

SCHOLARONE™
Manuscripts

I, the Submitting Author has the right to grant and does grant on behalf of all authors of the Work (as defined in the below author licence), an exclusive licence and/or a non-exclusive licence for contributions from authors who are: i) UK Crown employees; ii) where BMJ has agreed a CC-BY licence shall apply, and/or iii) in accordance with the terms applicable for US Federal Government officers or employees acting as part of their official duties; on a worldwide, perpetual, irrevocable, royalty-free basis to BMJ Publishing Group Ltd ("BMJ") its licensees and where the relevant Journal is co-owned by BMJ to the co-owners of the Journal, to publish the Work in this journal and any other BMJ products and to exploit all rights, as set out in our licence.

The Submitting Author accepts and understands that any supply made under these terms is made by BMJ to the Submitting Author unless you are acting as an employee on behalf of your employer or a postgraduate student of an affiliated institution which is paying any applicable article publishing charge ("APC") for Open Access articles. Where the Submitting Author wishes to make the Work available on an Open Access basis (and intends to pay the relevant APC), the terms of reuse of such Open Access shall be governed by a Creative Commons licence – details of these licences and which Creative Commons licence will apply to this Work are set out in our licence referred to above.

Other than as permitted in any relevant BMJ Author's Self Archiving Policies, I confirm this Work has not been accepted for publication elsewhere, is not being considered for publication elsewhere and does not duplicate material already published. I confirm all authors consent to publication of this Work and authorise the granting of this licence.

For peer review only

Lack of acculturation does not explain excess COVID-19 mortality among immigrants. A population-based cohort study

Siddartha Aradhya[1], Maria Brandén[1,2], Sven Drefahl[1], Ognjen Obućina[3], Gunnar Andersson[1], Mikael Rostila[4,5], Eleonora Mussino[1], Sol P. Juárez[4,5*]

1. Stockholm University Demography Unit, Stockholm University, 106 91 Stockholm, Sweden

2. Institute for Analytical Sociology, Linköping University, 601 74 Norrköping, Sweden

3. Institut national d'études démographiques (INED), 9 cours des Humanités, 93322 Aubervilliers, France

4. Department of Public Health Sciences, Stockholm University, 106 91 Stockholm, Sweden

5. Centre for Health Equity Studies (CHESS), Stockholm University/Karolinska Institute, Stockholm, Sweden

[*] Corresponding author: Sol P. Juárez, sol.juarez@su.se Department of Public Health Sciences, Stockholm University, SE-106 91, Stockholm, Sweden

Keywords: COVID-19; immigrants; acculturation; mortality; Sweden

Word count. 2964

For peer review only - http://bmjopen.bmj.com/site/about/guidelines.xhtml

Lack of acculturation does not explain excess COVID-19 mortality among immigrants. A population-based cohort study

Abstract

Objectives. To examine how excess mortality from COVID-19 among immigrants is associated with level of acculturation.

Design. Cohort study with follow-up between March 4 and May 7, 2020.

Setting. Swedish register-based study on all residents in Stockholm County.

Participants. 836,390 Stockholm residents in co-residential unions who were 30 years of age or older and alive on March 4th, 2020 and living in Sweden in December 2019.

Outcome measures. Cox regression models were conducted to assess the association between different constellations of immigrant-native couples (measure of acculturation) and COVID-19 mortality and all other causes of deaths (2019 and 2020). All other causes of death were used to contrast how acculturation is associated with other causes of death. Models were adjusted for relevant confounders.

Results. Compared to Swedish-Swedish couples (3.03 deaths per thousand person years), both immigrants partnered with another immigrant and a native showed excess mortality for COVID-19 (HR 1.45; 95%CI 1.12, 1.88 and HR 1.53; 95%CI 1.15, 2.05, respectively) which translates to 4.43 and 4.24 deaths per thousand person years. Moreover, similar results are found for natives partnered with an immigrant (HR 1.39; 95%CI 1.03, 1.88) which translates to 3.94 deaths per thousand person years. Further analysis shows that immigrants from both high and low-middle income countries experience similar levels of excess mortality when partnered with a Swede. Moreover, patterns of mortality for all other causes of death show that immigrants, on average, have the same or lower mortality than natives regardless of the origin of their partner.

Conclusions. Immigrants experience excess mortality relative to Swedes from COVID-19 across levels of acculturation. Public health strategies based on cultural differences might not only be inefficient but also reinforce stereotypes and heath inequalities.

For peer review only - http://bmjopen.bmj.com/site/about/guidelines.xhtml

Article Summary

- This study uses population-register data with complete coverage of the total population and all deaths (from COVID-19 and other causes) in the Stockholm region.
- Using population data linked to various registers we were able to identify the origin of co-resident couples (a well-established indicator of acculturation) to evaluate some of the hypotheses given to immigrants' excess Covid-19 mortality in Sweden (e.g., language barrier, unawareness of the public health recommendations and/or trust in institutions).
- Our analytical approach reveals that excess COVID-19 mortality among immigrants is irrespective of the partner's origin but also that Swedish-born individuals (without immigrant background) living with immigrants also experienced excess mortality compared to their native counterparts living with other Swedes. These findings allow the debate to move beyond the dichotomy immigrants vs. natives.
- Our study controls for alternative explanations such as education, income, housing conditions, and neighbourhood immigrant density. However, it lacks occupation information.

For peer review only

For peer review only - http://bmjopen.bmj.com/site/about/guidelines.xhtml

International evidence has shown that immigrants and ethnic minorities are disproportionately at risk of severe COVID-19 complications and death[1–8]. In the context of an ongoing pandemic, an effort to understand the causes for why some groups are more affected is a public health priority[9–12]. In the case of immigrants and ethnic minorities, labor market segregation, poor living conditions, underlying health, genetic predisposition, and healthcare access have been listed as potential explanations[2,4,11–14]. Recent studies, however, suggest that immigrants and minorities maintain an excess mortality even after controlling for socio-economic status and housing conditions[1,3]. As a result, a separate explanation has surfaced, with little empirical support, that cultural differences including, but not limited to, frequency of intergenerational contact, lifestyles, lower adherence to health recommendations, and underutilization of health care services are factors that lead immigrant groups to be more severely affected by the virus[2,4,10,12].

The role of culture has also manifested itself in some of the national strategies adopted to fight against the pandemic. Sweden, for example, has justified implementing a relatively less rigid approach by arguing that Swedes have a high level of trust in their institutions and as such follow governmental recommendations[15,16]. The strategy relied primarily on public health advice (regarding hygiene routines, social distancing and suspension from work, school or daycare in case of minor symptoms) *in lieu* of mandates which are not permitted under Swedish law[15]. The effectiveness of the adopted strategy relies on the assumption that the Swedish society will homogeneously change their social behavior. Under this rationale, it is unsurprising that excess mortality observed among immigrants[17]—especially concentrated among those with more distant origins—could be interpreted as a consequence of less acculturation and/or related factors. This reasoning can be used, in turn, to reinforce anti-immigration, xenophobic sentiments, and stereotypes that have health consequences beyond the present pandemic[18].

The underlying assumption is based on the idea that natives (in this case ethnic Swedes) have certain behaviors (e.g., language, lifestyle, family contact, norms, and understanding of the healthcare system) that make them less vulnerable to COVID-19, whereas, immigrants to a large extent lack these characteristics. Extending this reasoning

For peer review only - http://bmjopen.bmj.com/site/about/guidelines.xhtml

to the household level, one can assume that immigrants partnered with Swedes share and have access to such protective factors making them less vulnerable (i.e., most acculturated group); whereas, immigrants partnered with other immigrants are more likely to entirely lack access to such protective factors (i.e., less acculturated group). If the explanation that less acculturation explains excess mortality of immigrants is valid, one may expect that immigrants will experience lower mortality if they are partnered with a Swede versus another immigrant. To this end, the aim of this study is to examine the association between acculturation and COVID-19 mortality by examining native-immigrant couple dyads—a well-regarded measure that has been shown to be a marker and facilitator of acculturation[19–21].

Methods

Study population

An observational cohort study was conducted using Swedish register data. The study includes all Stockholm residents who were 30 years of age or older and were cohabiting with another adult who was at least 30 years of age and alive on March 4th, 2020, residing in Sweden in December 2019 (n=853,376). This age restriction was established to ensure cohabiting individuals were family members and not flat mates. The follow-up period was March 4 up until May 7, 2020. We excluded individuals who had not lived in Sweden in the two prior years (n=11,864) and those with missing data on country of birth of either partner (n=35) and income of either partner (n=5,087). The final study population consists of 836,390 individuals (26.5% immigrants) (figure 1).

Patient and public involvement

No patient involved

Data

We use information from several Swedish administrative registers linked through personal identity numbers that are unique to each person with legal residence in Sweden. Data on deaths were retrieved from the Cause of Death Register. Socioeconomic and demographic variables (income, education, and number of children) were drawn from the

For peer review only - http://bmjopen.bmj.com/site/about/guidelines.xhtml

Longitudinal integrated database for health insurance and labor market studies (LISA), whereas residential information (type and size of dwelling and immigrant density in the neighborhood) were drawn from the Dwelling register. All covariates in our study are time-constant and either measured at the end of 2019 (all variables at the household and neighborhood level) or 2018 (highest education attained, sum of the individual net incomes of the two co-resident adults, total number of individuals in the household under 30). Information on age, sex, and country of birth stem from the Total Population Register. It is important to note that all individuals registered in Sweden are entitled to health care access.

Study variables

COVID-19 mortality was identified by the Swedish National Board of Health and Welfare, the agency responsible for the cause of death register. COVID-19 mortality was identified using the following ICD codes: 490 cases had emergency ICD code U07.1, U07.2 or B342; in 25 cases U07.1, U07.2 or B342 were listed as contributing causes of death, excluding mortality from other causes of death (1,072 cases). Given the timeliness of the data, the assignment of the underlying cause of death should be understood as preliminary.

Immigrant-native couple types were created by combining information from the dwelling and the total population register to create the couple type which include two individuals of at least 30 years of age co-residing in the same household. The variable is classified into the following four ego-partner categories using information on country of birth: (i) native-native, (ii) native-immigrant, (iii) immigrant-native, and (iv) immigrant-immigrant. We chose (i) native-native couples as the reference group for our analyses as they a) constitute the largest category among all groups considered and b) represent the culture and language of the host population from which we expect other groups to deviate. We further disaggregated the groups by immigrant's origins defined according to the World Bank classification based on the Gross National Incomes (GNI) per capita using the WB Atlas method[22] as low-middle and high income countries.

For peer review only - http://bmjopen.bmj.com/site/about/guidelines.xhtml

We derived individual income and calculated the sum of the two partners' net incomes, categorized into tertiles based on all adult residents of Sweden. We derived education data from Swedish educational registers and categorize our population into four categories; those with primary schooling, secondary schooling, post-secondary education, and those with missing information on education. Missing information on education is generally very low but 88% of those with missing education are immigrants. From the Swedish Dwelling register we accessed information on size of the dwelling and a unique dwelling code which enables us to link individuals who live together in a household and determine co-residence. From this information we create: the number of individuals per square meter in the household (with a separate category for a small group of individuals, due to missing information on square meters in some detached houses), and the number of individuals living in the household. We include in our model also the share of immigrants in the local neighborhood, DeSO (a smaller subdivision in Swedish administrative statistics).

Statistical analysis

We conducted Cox proportional hazards regressions (using age as the timescale) to estimate Hazard Ratios (HR) and 95% Confidence Intervals (CI) for the association between immigrant-native couple type and COVID-19 mortality. Individuals exited the study by (1) dying between March 5, 2020 and May 7, 2020, or (2) being alive on May 7, 2020. We estimated two separate regressions estimating the cause-specific hazard of dying from COVID-19, right-censoring all individuals that die from other causes and (2) the cause-specific hazard of dying from other causes than COVID-19, right-censoring all individuals that die from COVID-19. In addition, we conducted Cox regressions for dying from all causes of death that occurred between March 5, 2019 and May 7, 2019 (981 deaths), the same time of the year we observe COVID-19 deaths in 2020. Since mortality from COVID-19 and other causes of deaths in 2020 are not fully independent of each other, our estimates for all-cause mortality in 2019 were used to evaluate the robustness of our 2020 estimates. In addition, the comparison between all-cause mortality in 2019 and mortality in 2020 by level of acculturation will allow us to examine whether the latter has a distinctive role in relation to the pandemic.

For peer review only - http://bmjopen.bmj.com/site/about/guidelines.xhtml

Two models were estimated: 1) a simple model with age as the time scale adjusted for sex and 2) the same model with further adjustments. In the latter analysis we adjust for education and neighborhood characteristics that are confounders, as well as factors that are on the causal pathway that have been previously used to explain the excess mortality of immigrants (i.e, number of individuals in the household below the age of 30, dwelling type, square meters per person in the dwelling, household income). In addition, we conducted two sensitivity analyses. One in which we examined the partner's origin in the Swedish-migrant partnership (high and low-middle income) (Appendix figure 1) and, a second, in which we excluded all individuals born in Sweden with at least one foreign-born parent and the results remain unchanged (Appendix figure 2). All analyses were conducted using Stata Statistical Software: Release 16 (StataCorp LP, College Station, Texas).

This study was approved by the Central Ethical Review Board in 2020 (Dnr 2020-02199).

Results

During the 149,622 person-years of observation, 1,587 deaths occurred in our study population between March 5, 2020 and May 7, 2020. Table 1 shows the distribution of population at risk and deaths by all covariates. In our population, 17.7% of individuals are in immigrant-native couples. Native-native couples show the lowest deaths per thousand person years (3/1000), whereas all other couple types show higher death rates of COVID-19 mortality (approximately 4 per thousand person-years). Of the study population, 20.8% of COVID-19 deaths were attributed to native-immigrant mixed couples and 22.7% to immigrant-immigrant couples.

Figure 2 displays mortality risks from COVID-19, all other causes of death in 2020, and all-causes of death in 2019 across couple types with native-native as the reference. Panel A presents models adjusted for age and sex, panel B presents the estimates including adjustments. In A, individuals in immigrant-immigrant couples show the highest HR of

For peer review only - http://bmjopen.bmj.com/site/about/guidelines.xhtml

dying from COVID-19 (HR 2.51; 95%CI: 2.02, 3.12) and those in native-native couples the lowest (reference group) while and immigrants in immigrant-native couples showed intermediate mortality levels (HR: 1.76; 95%CI: 1.32, 2.36). All-causes of death in 2019 and 2020 show similar patterns with little differences between couple constellations. After adjustments (panel B) individuals in all couple types other than native-native couples display similar and higher HRs from COVID-19 mortality, but almost no differences in HRs across groups in other causes of death in 2020 and all-causes of death in 2019. An excess mortality was also observed for natives in native-immigrant couples (HR 1.60; 95%CI: 1.19, 2.15) that remained after adjustment (HR 1.39; 95%CI: 1.03, 1.88).

Figure 3 is an extension of figure 1 disaggregating the immigrant population by income level of their country of birth. In panel A, there is elevated HRs across all origin groups relative to native-native couples. Individuals in LMIC-immigrant couples display the highest HR (HR 3.59; 95%CI 2.79, 4.63), whereas individuals in all other couple types with at least one immigrant display relatively similar HRs (around 1.5). After adjustment (panel B), all groups with an immigrant still display higher HRs relative to native-native couples, but at a lower level. LMIC-immigrant couples experience a particularly strong reduction in their HRs (HR 1.82; 95%CI 1.31, 2.52). Compared to LMIC-native couples, their HR remains slightly higher (HR 1.56; 95%CI 0.80, 3.06). We also find HIC-native (HR 1.55; 95%CI 1.13, 2.12) and HIC-immigrant (HR 1.15; 95%CI 0.81, 1.64) couples display higher HRs relative to the reference group.

Discussion

Our study shows that being partnered with a Swedish-born person is not protective against COVID-19 mortality for immigrants. In fact, they display the same or higher levels of mortality from COVID-19 when partnered with a Swede as when partnered with another immigrant. These findings challenge the hypothesis that factors related to the familiarity and closeness to the host society, such as lack of awareness of recommendations, language barriers, and access to information are relevant factors when explaining the excess mortality from COVID-19 among immigrants. Importantly, we show that this pattern holds across immigrant groups (HIC and LMIC). The comparison between couple

For peer review only - http://bmjopen.bmj.com/site/about/guidelines.xhtml

types with respect to mortality from all other causes in 2020 and all-cause mortality in 2019 strengthens our findings as it demonstrates that the excess mortality from COVID-19 is observed in couples that show no excess mortality from other causes before and during the pandemic.

Language has been considered a vital component of acculturation and relevant for accessing information on other types of medical treatments and health outcomes[23]. Although we cannot test this aspect directly, our study shows the lack of language barriers do not seem to be protective for the most acculturated group (immigrants partnered with a Swede), which raises the question as to whether language barriers explain at all the excess mortality among immigrants partnered with another immigrant. Given that the COVID-19 pandemic is a unique occurrence that was accompanied by global diffusion of information, one can argue that even the least acculturated (immigrants cohabiting with an immigrant) have been exposed to recommendations offered in their native languages from either public health officials from their countries of origin or via other international channels. In fact, the information that they may have received from international sources may be more relevant for specific immigrant populations, for example, how to best protect oneself when observing cultural or religious practice. Prior studies in clinical settings have shown that culturally adapted information is associated with better health access and outcomes[23].

Beyond allowing us to disentangle the role of acculturation and language barriers in the excess COVID-19 mortality among immigrants, our study provides suggestive evidence with respect to genetic arguments. Our results suggest that 'genetic predisposition and/or differences in susceptibility or response to infection'[12] plays no visible role in explaining the excess COVID-19 mortality among immigrants. Although it is true that Swedes partnered with a Swede show the lowest mortality, those partnered with an immigrant have the same level of COVID-19 mortality as the immigrants themselves. Given that Swedes partnered with an immigrant have the same level of all-cause mortality as Swedes partnered with a Swede (yet high levels of COVID-19 mortality), it seems unlikely that genetic selection into family types matter in these groups. At the same time, there is

no evidence that immigrants who partnered with a Swede are inherently different from those who partnered with an immigrant.

In this study, immigrants in different family constellations show higher levels of COVID-19 deaths than the majority of natives, after adjustment for a wide range of individual- and contextual-level factors, including education, income, housing conditions, and neighborhood immigrant density. This set of adjustments also partly accounts for a number of socially patterned chronic health conditions and comorbidities, e.g., insulin resistance, hypertension, smoking and obesity, which have been suggested as risk factors for severe cases of COVID-19[12].

Further research should disentangle why Swedes partnered with immigrants experience higher mortality than those partnered with Swedes. Appendix figure 1 shows that Swedes have equally elevated mortality relative to those partnered with other Swedes regarding of the origin of the immigrant partner (HICs or LMICs). This finding suggests that the negative effect of living with an immigrant (if it exists) is not related to more traditional patterns of family structure or participation in very different cultural practices.

This study is the first to examine acculturation as the mechanism behind the disproportionate burden that COVID-19 has placed on immigrant communities, by comparing the mortality of native-native, immigrant-native, and immigrant-immigrant couples in Stockholm, Sweden. This is an ideal setting for our study due to its heterogeneous immigrant population that represent a substantive share of the total Swedish population. A major strength of our study is that we have complete coverage of the total population and all deaths in the Stockholm region, from both COVID-19 and all other causes of deaths. Thus, our analysis does not suffer from selection into our study population. We have similar high-quality data for 2019, which allows for an unbiased comparison of mortality patterns between the two years. Although the Swedish population registers hold high quality and have many advantages, they capture de jure rather than de facto characteristics of individuals. With respect to our measure of partnership, 79% of the couples in our data are either married or have shared children while the remaining 21% co-reside unmarried without common children. However, non-marital cohabitation is

For peer review only - http://bmjopen.bmj.com/site/about/guidelines.xhtml

very common in Sweden, while flat-sharing is not[25], and particularly among individuals above 30s. It is therefore fair to assume that a substantive share in this remaining group is cohabiting in an amorous relationship without common children, a group that is often overlooked in international studies of health and mortality because of the lack of data.

A final limitation is related to a lack of information regarding occupation. Specifically, it has been hypothesized that immigrants are more likely to work in ‘front-line’ occupations; however, a majority of deaths are occurring among retired individuals with no attachment to the labor market.

In conclusion, our study shows that being partnered with a native is neither protective for immigrants nor contributes to closing the gap with natives, even after adjusting for a wide range of possible confounders on both individual- couple- and residential level. As such, these findings show that poor level of acculturation, unawareness of the Swedish recommendations and language barriers do not explain the excess COVID-19 mortality of immigrants. At the same time, however, Swedes partnered with immigrants also show equal excess mortality compared to Swedish couples. Therefore, our study shows that focusing on simple native-immigrant dichotomies is not only oversimplified but may reinforce ethnic stereotypes and health inequalities.

For peer review only - http://bmjopen.bmj.com/site/about/guidelines.xhtml

References:

1. Drefahl, S. *et al.* A population-based cohort study of socio-demographic risk factors for COVID-19 deaths in Sweden. *Nat Commun* **11**, 5097 (2020).
2. Aldridge, R. W. *et al.* Black, Asian and Minority Ethnic groups in England are at increased risk of death from COVID-19: indirect standardisation of NHS mortality data. *Wellcome Open Res* **5**, 88 (2020).
3. Rostila, M. *et al.* Disparities in covid-19 deaths by country of birth in Stockholm, Sweden: A total population based cohort study. *Stockholm Research Reports in Demography. Preprint.* (2020) doi:https://doi.org/10.17045/sthlmuni.12852854.v1.
4. Niedzwiedz, C. L. *et al.* Ethnic and socioeconomic differences in SARS-CoV-2 infection: prospective cohort study using UK Biobank. *BMC Med* **18**, 160 (2020).
5. Ross, J., Diaz, C. M. & Starrels, J. L. The Disproportionate Burden of COVID-19 for Immigrants in the Bronx, New York. *JAMA Intern Med* (2020) doi:10.1001/jamainternmed.2020.2131.
6. Millett, G. A. *et al.* Assessing Differential Impacts of COVID-19 on Black Communities. *Annals of Epidemiology* S1047279720301769 (2020) doi:10.1016/j.annepidem.2020.05.003.
7. Pan, D. *et al.* The impact of ethnicity on clinical outcomes in COVID-19: A systematic review. *EClinicalMedicine* **23**, 100404 (2020).
8. Williamson, E. J. *et al.* Factors associated with COVID-19-related death using OpenSAFELY. *Nature* **584**, 430–436 (2020).
9. Statement by the EUPHA Migrant and ethnic minority health section on COVID-19 – CALL FOR ACTION: Reducing the impact of the coronavirus crisis on those ‘left behind’ – Disadvantaged Migrants and Ethnic Minorities (MEM). (2020).

For peer review only - http://bmjopen.bmj.com/site/about/guidelines.xhtml

10. Pareek, M. *et al.* Ethnicity and COVID-19: an urgent public health research priority. *The Lancet* **395**, 1421–1422 (2020).
11. Bhala, N., Curry, G., Martineau, A. R., Agyemang, C. & Bhopal, R. Sharpening the global focus on ethnicity and race in the time of COVID-19. *The Lancet* **0**, (2020).
12. Khunti, K., Singh, A. K., Pareek, M. & Hanif, W. Is ethnicity linked to incidence or outcomes of covid-19? *BMJ* **369**, (2020).
13. Hooper, M. W., Nápoles, A. M. & Pérez-Stable, E. J. COVID-19 and Racial/Ethnic Disparities. *JAMA* (2020) doi:10.1001/jama.2020.8598.
14. Guadagno, L. *Migrants and the COVID-19 pandemic: An initial analysis*. 28 (2020).
15. Pierre, J. Nudges against pandemics: Sweden's COVID-19 containment strategy in perspective. *Policy and Society* **39**, 478–493 (2020).
16. Paterlini, M. 'Closing borders is ridiculous': the epidemiologist behind Sweden's controversial coronavirus strategy. *Nature* **580**, 574–574 (2020).
17. Folkhälsomydigheten. *Veckorapport om covid-19, vecka 15*. https://www.folkhalsomyndigheten.se/globalassets/statistik-uppfoljning/smittsamma-sjukdomar/veckorapporter-covid-19/2020/covid-19-veckorapport-vecka-15.pdf (2020).
18. Le, T. K., Cha, L., Han, H.-R. & Tseng, W. Anti-Asian Xenophobia and Asian American COVID-19 Disparities. *Am J Public Health* **110**, 1371–1373 (2020).
19. Alba, R. D. & Golden, R. M. Patterns of Ethnic Marriage in the United States. *Social Forces* **65**, 202–223 (1986).

For peer review only - http://bmjopen.bmj.com/site/about/guidelines.xhtml

20. Lieberson, S. & Waters, M. C. Ethnic groups in flux: the changing ethnic responses of American whites. *The Annals of the American Academy of Political and Social Science* **487**, 79–91 (1986).

21. Kalmijn, M. Intermarriage and Homogamy: Causes, Patterns, Trends. *Annual Review of Sociology* **24**, 395–421 (1998).

22. The World Bank. GNI, Atlas method (current US$) | Data. *DataBank* https://data.worldbank.org/indicator/NY.GNP.ATLS.CD.

23. Flores, G. The Impact of Medical Interpreter Services on the Quality of Health Care: A Systematic Review. *Med Care Res Rev* **62**, 255–299 (2005).

24. Pollán, M. *et al.* Prevalence of SARS-CoV-2 in Spain (ENE-COVID): a nationwide, population-based seroepidemiological study. *The Lancet* S0140673620314835 (2020) doi:10.1016/S0140-6736(20)31483-5.

25. SCB. *Ensam, med partner eller kompisboende Vad säger hushållsregistret?* https://www.scb.se/contentassets/cfe7690018d741798939bd8a6d087219/be0701_2015i2018_br_be51br1901.pdf (2019).

26. Billingsley, S. *et al.* Deaths in the frontline: Occupation-specific COVID-19 mortality risks in Sweden. *Stockholm Research Reports in Demography. Preprint.* (2020) doi:DOI: 10.17045/sthlmuni.12816065.v2.

For peer review only

For peer review only - http://bmjopen.bmj.com/site/about/guidelines.xhtml

Table 1. Description of the study population, number, proportion of deaths and death rates

	Total		COVID-19		Other cause of death 2020		Exposure time in years	COVID-19 deaths per 1000 years
	N at March 5	%	Deaths March 5 – May 7	%	Deaths March 5 – May 7	%		
**Couple type**								
Native-native	54,0515	64.6	291	56.5	748	69.8	96,101	3.03
Native-immigrant	74,247	8.9	52	10.1	88	8.2	13,201	3.94
Immigrant-native	72,989	8.7	55	10.7	82	7.6	12,979	4.24
Immigrant-immigrant	148,639	17.8	117	22.7	154	14.4	26,431	4.43
**Couple type, detailed**								
Native-native	540,515	64.6	291	56.5	748	69.8	96,101	3.03
Native-immigrant	74,247	8.9	52	10.1	88	8.2	13,201	3.94
HIC-native	40,795	4.9	46	8.9	72	6.7	7,251	6.34
HIC-immigrant	39,950	4.8	37	7.2	58	5.4	7,103	5.21
LMIC-native	32,194	3.8	9	1.7	10	0.9	5,728	1.57
LMIC-immigrant	108,689	13.0	80	15.5	96	9.0	19,328	4.14
**Sex**								
Male	421,266	50.4	342	66.4	677	63.2	74,885	4.57
Woman	415,124	49.6	173	33.6	395	36.8	73,828	2.34
**Education**								
Primary	91,205	10.9	143	27.8	305	28.5	16,194	8.83
Secondary	293,506	35.1	208	40.4	441	41.1	52,178	3.99
Post-secondary	440,907	52.7	136	26.4	302	28.2	78,427	1.73
Missing	10,772	1.3	28	5.4	24	2.2	1,913	14.64
**Household income (tertile)**								
Lowest	204,497	24.4	349	67.8	659	61.5	36,308	9.61
Middle	226,798	27.1	96	18.6	242	22.6	40,333	2.38
Highest	405,095	48.4	70	13.6	171	16.0	72,071	0.97
**Housing type**								
Multi-family	452,305	54.1	314	61.0	640	59.7	80,412	3.90
Single-family	379,232	45.3	142	27.6	359	33.5	67,447	2.11
Care home	4,853	0.6	59	11.5	73	6.8	853	69.19
**Number of people under 30 in the household**								
0	379,111	45.3	449	87.2	964	89.9	67,348	6.67
1	151,330	18.1	39	7.6	48	4.5	26,924	1.45
2	213,803	25.6	17	3.3	42	3.9	38,044	0.45
3+	92,146	11.0	10	1.9	18	1.7	16,396	0.61
**m2/person in the household (crowdedness)**								
0-	106,763	12.8	56	10.9	80	7.5	18,988	2.95
20-	230,997	27.6	72	14.0	149	13.9	41,089	1.75
30-	199,904	23.9	148	28.7	291	27.1	35,539	4.16
40-	194,915	23.3	153	29.7	341	31.8	34,646	4.42
60-	97,904	11.7	85	16.5	205	19.1	17,399	4.89
Missing	5,907	0.7	1	0.2	6	0.6	1,051	0.95

For peer review only - http://bmjopen.bmj.com/site/about/guidelines.xhtml

For peer review only

**Share immigrants in DeSO (%)**								
0-	51,045	6.1	17	3.3	58	5.4	9,078	1.87
0.10-	205,426	24.6	70	13.6	232	21.6	36,531	1.92
0.15-	235,209	28.1	127	24.7	261	24.3	41,825	3.04
0.20-	158,718	19.0	115	22.3	216	20.1	28,218	4.08
0.30-	117,886	14.1	91	17.7	195	18.2	20,955	4.34
0.50-	68,106	8.1	95	18.4	110	10.3	12,104	7.85
**TOTAL**	836,390	100.0	515	100.0	1072	100.0	148,712	3.46

For peer review only

For peer review only - http://bmjopen.bmj.com/site/about/guidelines.xhtml

Figure legends.

Figure 1. Selection flow and final sample

Figure 2. Hazard Ratios for (A) adjusted for age and sex only and (B) further adjusted associations between immigrant-native couple type (reference group: native-native couples), COVID-19 and all-cause mortality in 2020 in Stockholm, Sweden.

Figure 3. Hazard Ratios for (A) adjusted for age and sex only and (B) further adjusted associations between immigrant-native couple type for specific country groups (reference group: native-native couples), COVID-19 mortality and mortality from all other causes in 2020 and 2019 in Stockholm, Sweden.

For peer review only

For peer review only - http://bmjopen.bmj.com/site/about/guidelines.xhtml

Figure 1. Selection flow and final sample

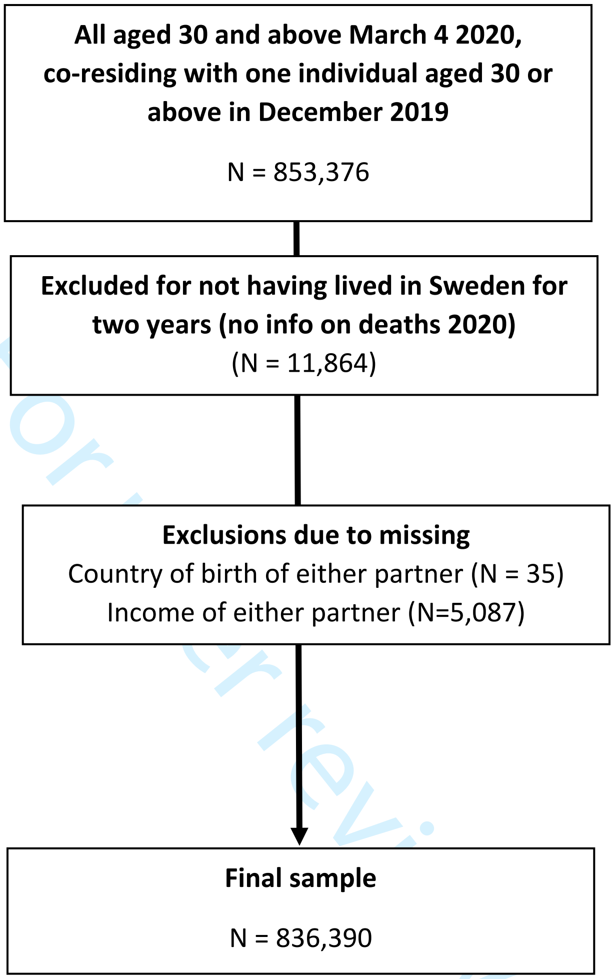

For peer review only - http://bmjopen.bmj.com/site/about/guidelines.xhtml

Figure 2. Hazard Ratios for (A) adjusted for age and sex only and (B) further adjusted associations between immigrant-native couple type (reference group: native-native couples), COVID-19 and all-cause mortality in 2020 in Stockholm, Sweden.

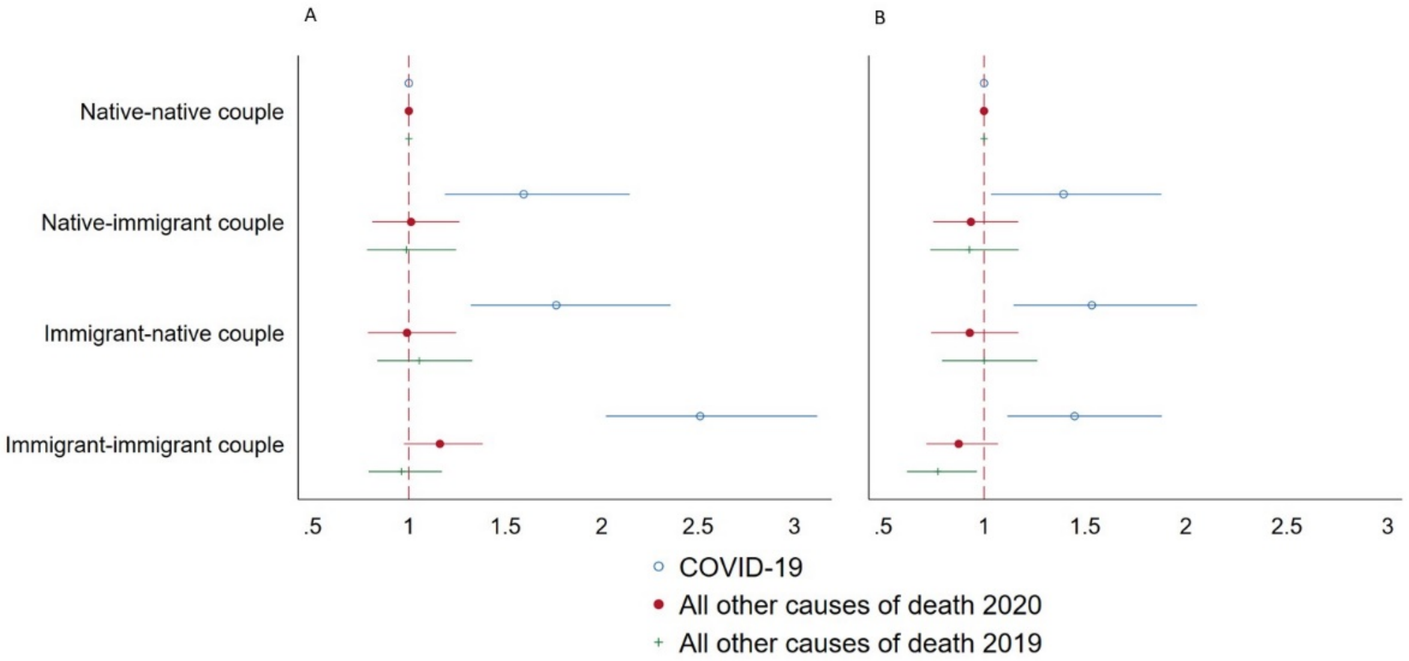

Figure 3. Hazard Ratios for (A) adjusted for age and sex only and (B) further adjusted associations between immigrant-native couple type for specific country groups (reference group: native-native couples), COVID-19 mortality and mortality from all other causes in 2020 and 2019 in Stockholm, Sweden.

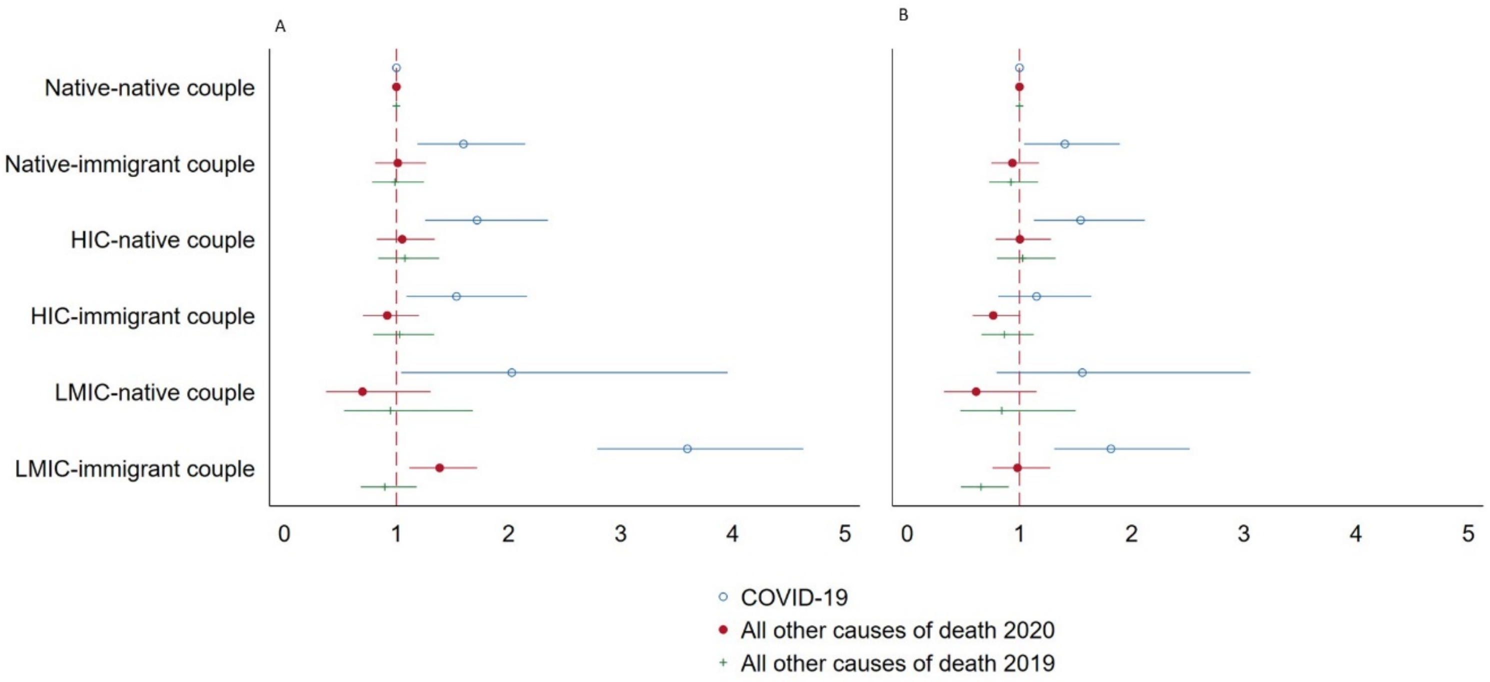

Note: LMIC= Low and middle income countries; HIC=High income countries

Supplementary material

Appendix Figure 1: Sensitivity analysis disaggregating Native-immigrant partnerships by immigrant partners' origin

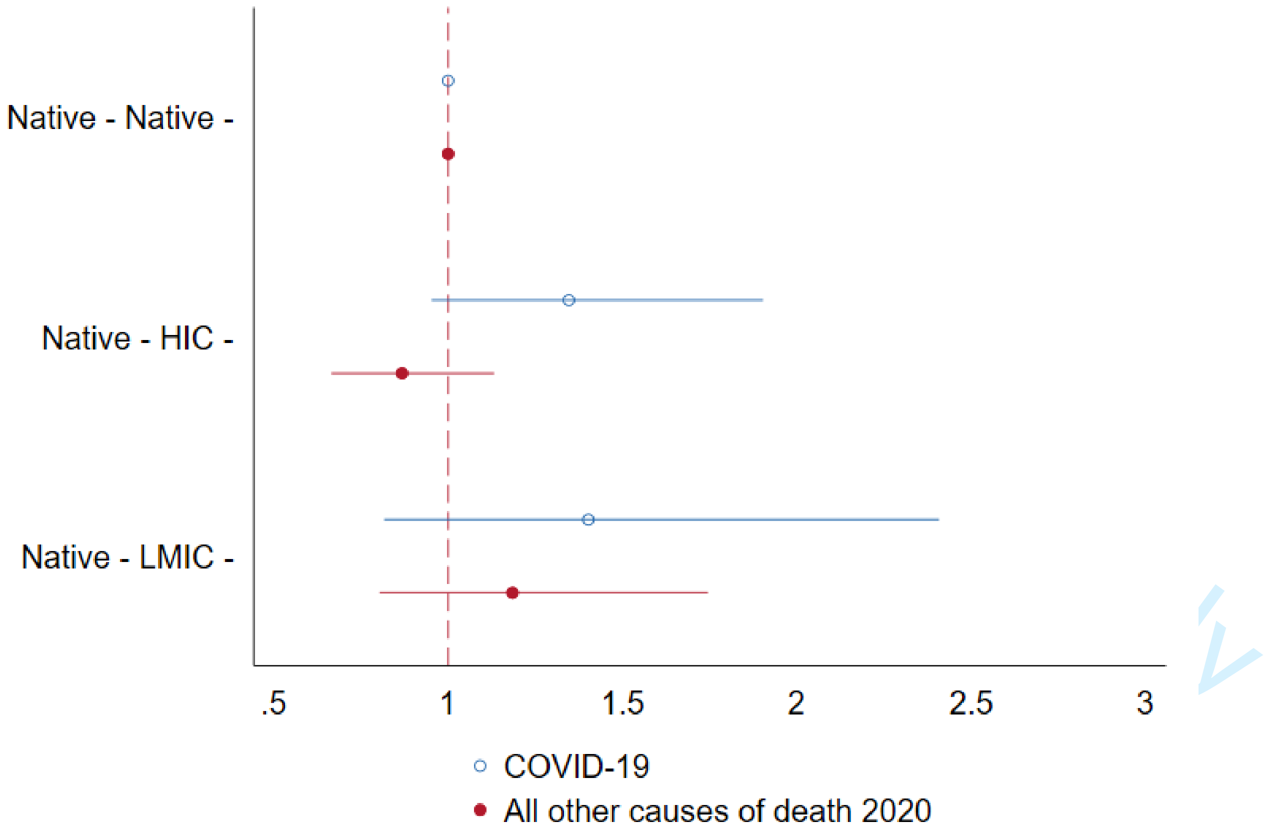

Note: LMIC= Low and middle income countries; HIC=High income countries

Appendix Figure 2: Sensitivity analysis of figure 2B excluding second generation immigrants

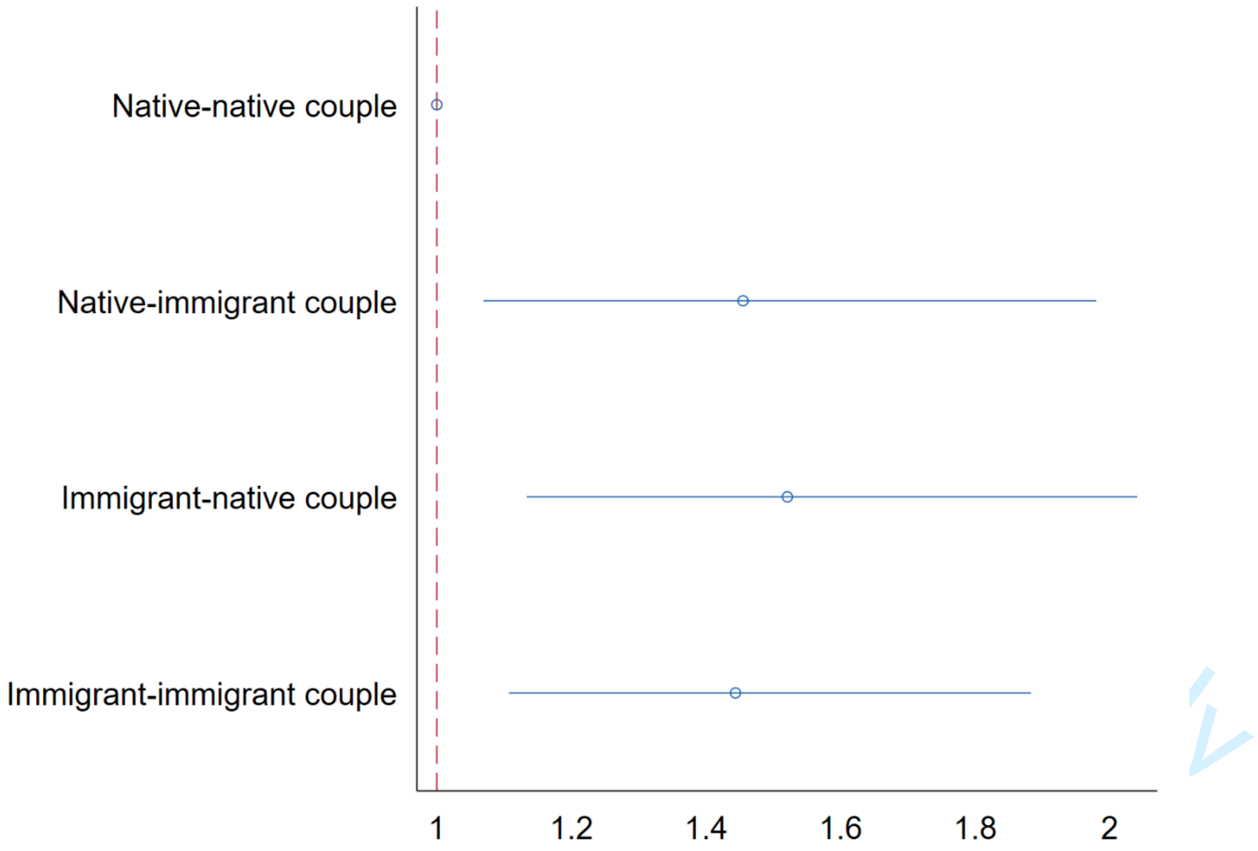

Appendix table 1: Full regression table for Figure 2

	Model 1: Figure 2A						Model 2: Figure 2B					
	COVID- 19		Other COD 2020		All COD 2019		COVID- 19		Other COD 2020		All COD 2019	
	HR	95% CI	HR	95% CI	HR	95% CI	HR	95% CI	HR	95% CI	HR	95% CI
**Partnership type**												
Native-native	1.00	1.00	1.00	1.00	1.00	1.00	1.00	1.00	1.00	1.00	1.00	1.00
Native-immigrant	1.60	(1.19, 2.15)	1.01	(0.81, 1.26)	0.99	(0.78, 1.24)	1.39	(1.03,1.88)	0.94	(0.75,1.17)	0.93	(0.73,1.17)
Immigrant-native	1.76	(1.32, 2.36)	0.99	(0.79, 1.25)	1.05	(0.84, 1.33)	1.53	(1.15,2.05)	0.93	(0.74,1.17)	1.00	(0.79,1.26)
Immigrant-immigrant	2.51	(2.02, 3.12)	1.16	(0.97, 1.38)	0.96	(0.79, 1.17)	1.45	(1.12,1.88)	0.87	(0.71,1.07)	0.77	(0.62,0.96)
Years under risk	148,712.19		148,712.19		148,268.51		148,712.19		148,712.19		148,268.51	
N events	518		1072		981		518		1072		981	
N	836390		836390		833650		836390		836390		833650	

Model 1 includes adjustments for sex; Model 2 includes adjustments for sex, household income, education, housing type, number of individuals in the household, m^2/person in the household (crowdedness), share of immigrants in the neighborhood of residence

For peer review only

Appendix table 2: Full regression table for Figure 3

	Model 3: Figure 3A						Model 4: Figure 3B					
	COVID- 19		Other COD 2020		All COD 2019		COVID- 19		Other COD 2020		All COD 2019	
	HR	95% CI	HR	95% CI	HR	95% CI	HR	95% CI	HR	95% CI	HR	95% CI
**Partnership type**												
Native-native	1.00	1.00	1.00	1.00	1.00	1.00	1.00	1.00	1.00	1.00	1.00	1.00
Native-immigrant	1.60	(1.19,2.15)	1.01	(0.81,1.26)	0.99	(0.78,1.24)	1.40	(1.04,1.89)	0.94	(0.75,1.17)	0.92	(0.73,1.17)
HIC-native	1.72	(1.26,2.35)	1.05	(0.82,1.34)	1.08	(0.84,1.38)	1.55	(1.13,2.12)	1.00	(0.79,1.28)	1.03	(0.80,1.32)
HIC-immigrant	1.54	(1.09,2.16)	0.92	(0.70,1.20)	1.03	(0.79,1.34)	1.15	(0.81,1.64)	0.77	(0.58,1.01)	0.86	(0.66,1.13)
LMIC-native	2.03	(1.04,3.95)	0.70	(0.37,1.30)	0.95	(0.53,1.68)	1.56	(0.80,3.06)	0.61	(0.33,1.15)	0.84	(0.47,1.50)
LMIC-immigrant	3.59	(2.79,4.63)	1.38	(1.12,1.72)	0.90	(0.68,1.18)	1.82	(1.31,2.52)	0.98	(0.76,1.27)	0.66	(0.48,0.91)
Years under risk	148,712.19		148,712.19		148,268.51		148,712.19		148,712.19		148,268.51	
N events	518		1072		981		518		1072		981	
N	836,390		836,390		833,650		836,390		836,390		833,650	

Model 3 includes adjustments for sex; Model 4 includes adjustments for sex, household income, education, housing type, number of individuals in the household, m^2/person in the household (crowdedness), share of immigrants in the neighborhood of residence

For peer review only

STROBE Statement—checklist of items that should be included in reports of observational studies

	Item No	Recommendation
**Title and abstract**	1	(*a*) Indicate the study's design with a commonly used term in the title or the abstract **Yes**
		(*b*) Provide in the abstract an informative and balanced summary of what was done and what was found **Yes**
**Introduction**		
Background/rationale	2	Explain the scientific background and rationale for the investigation being reported **Page 5**
Objectives	3	State specific objectives, including any prespecified hypotheses **Page 5-6**
**Methods**		
Study design	4	Present key elements of study design early in the paper **Pages 6**
Setting	5	Describe the setting, locations, and relevant dates, including periods of recruitment, exposure, follow-up, and data collection **Page 6**
Participants	6	*Cohort study*—Give the eligibility criteria, and the sources and methods of selection of participants. Describe methods of follow-up **Pages 6 and page 8** *Case-control study*—Give the eligibility criteria, and the sources and methods of case ascertainment and control selection. Give the rationale for the choice of cases and controls *Cross-sectional study*—Give the eligibility criteria, and the sources and methods of selection of participants
		(*b*) *Cohort study*—For matched studies, give matching criteria and number of exposed and unexposed *Case-control study*—For matched studies, give matching criteria and the number of controls per case
Variables	7	Clearly define all outcomes, exposures, predictors, potential confounders, and effect modifiers. Give diagnostic criteria, if applicable **Pages 6-7**
Data sources/ measurement	8*	For each variable of interest, give sources of data and details of methods of assessment (measurement). Describe comparability of assessment methods if there is more than one group **Pages 6-7**
Bias	9	Describe any efforts to address potential sources of bias **Pages 8 and 9**
Study size	10	Explain how the study size was arrived at **Page 6 and figure 1**
Quantitative variables	11	Explain how quantitative variables were handled in the analyses. If applicable, describe which groupings were chosen and why **Pages 7-8**
Statistical methods	12	(*a*) Describe all statistical methods, including those used to control for confounding

For peer review only

Pages 8-9

(*b*) Describe any methods used to examine subgroups and interactions

Pages 8-9

(*c*) Explain how missing data were addressed

Figure 1, pages 8

(*d*) *Cohort study*—If applicable, explain how loss to follow-up was addressed

Page 8

Case-control study—If applicable, explain how matching of cases and controls was addressed

Cross-sectional study—If applicable, describe analytical methods taking account of sampling strategy

(*e*) Describe any sensitivity analyses

Continued on next page

For peer review only

**Results**		
Participants	13*	Report numbers of individuals at each stage of study—eg numbers potentially eligible, examined for eligibility, confirmed eligible, included in the study, completing follow-up, and analysed<br>**Not applicable**
		(b) Give reasons for non-participation at each stage<br>**Not applicable**
		(c) Consider use of a flow diagram<br>**Figure 1**
Descriptive data	14*	Give characteristics of study participants (eg demographic, clinical, social) and information on exposures and potential confounders<br>**Table 1, page 8, lines 218-225**
		(b) Indicate number of participants with missing data for each variable of interest<br>**Figure 1 and table 1**
		(c) *Cohort study*—Summarise follow-up time (eg, average and total amount)<br>**Page 8, lines 218-225, table 1**
Outcome data	15*	*Cohort study*—Report numbers of outcome events or summary measures over time<br>**page 8, lines 218-220, table 1**
		Case-control study—Report numbers in each exposure category, or summary measures of exposure
		Cross-sectional study—Report numbers of outcome events or summary measures
Main results	16	Give unadjusted estimates and, if applicable, confounder-adjusted estimates and their precision (eg, 95% confidence interval). Make clear which confounders were adjusted for and why they were included<br>**Figures 2 and 3**
		(*b*) Report category boundaries when continuous variables were categorized<br>**Table 1**
		(*c*) If relevant, consider translating estimates of relative risk into absolute risk for a meaningful time period
Other analyses	17	Report other analyses done—eg analyses of subgroups and interactions, and sensitivity analyses<br>**Page 7, lines 206-211 and page 11, lines 303-306**
**Discussion**		
Key results	18	Summarise key results with reference to study objectives<br>**Page 9-10, lines 254-265**
Limitations	19	Discuss limitations of the study, taking into account sources of potential bias or imprecision. Discuss both direction and magnitude of any potential bias<br>**Pages 11-12 lines 319-332**
Interpretation	20	Give a cautious overall interpretation of results considering objectives, limitations, multiplicity of analyses, results from similar studies, and other relevant evidence<br>**Pages 10-11, lines 267-308**
Generalisability	21	Discuss the generalisability (external validity) of the study results
**Other information**		
Funding	22	Give the source of funding and the role of the funders for the present study and, if applicable, for the original study on which the present article is based<br>**Yes**

For peer review only - http://bmjopen.bmj.com/site/about/guidelines.xhtml

*Give information separately for cases and controls in case-control studies and, if applicable, for exposed and unexposed groups in cohort and cross-sectional studies.

Note: An Explanation and Elaboration article discusses each checklist item and gives methodological background and published examples of transparent reporting. The STROBE checklist is best used in conjunction with this article (freely available on the Web sites of PLoS Medicine at http://www.plosmedicine.org/, Annals of Internal Medicine at http://www.annals.org/, and Epidemiology at http://www.epidem.com/). Information on the STROBE Initiative is available at www.strobe-statement.org.

For peer review only

BMJ Open

Intermarriage and COVID-19 mortality among immigrants. A population-based cohort study from Sweden

Journal:	*BMJ Open*
Manuscript ID	bmjopen-2021-048952.R1
Article Type:	Original research
Date Submitted by the Author:	18-May-2021
Complete List of Authors:	Aradhya, Siddartha ; Stockholms Universitet, Stockholm University Demography Unit<br>Branden, Maria; Linköping University, The Institute for Analytical Sociology (IAS); Stockholms Universitet, Stockholm University Demography Unit<br>Drefahl, Sven; Stockholm University, Sociology<br>Obucina, Ognjen; Institut national d'études démographiques<br>Andersson, Gunnar ; Stockholm University, Department of Sociology<br>Rostila, Mikael ; Stockholms Universitet, Department of Public Health Sciences<br>Mussino, Eleonora; Stockholms Universitet, Stockholm University Demography Unit<br>Juárez , Sol Pia; Stockholm University, Department of Public Health Sciences
**Primary Subject Heading**:	Public health
Secondary Subject Heading:	Global health, Public health, Sociology, Infectious diseases
Keywords:	COVID-19, PUBLIC HEALTH, Demography < TROPICAL MEDICINE, EPIDEMIOLOGY

SCHOLARONE™ Manuscripts

I, the Submitting Author has the right to grant and does grant on behalf of all authors of the Work (as defined in the below author licence), an exclusive licence and/or a non-exclusive licence for contributions from authors who are: i) UK Crown employees; ii) where BMJ has agreed a CC-BY licence shall apply, and/or iii) in accordance with the terms applicable for US Federal Government officers or employees acting as part of their official duties; on a worldwide, perpetual, irrevocable, royalty-free basis to BMJ Publishing Group Ltd ("BMJ") its licensees and where the relevant Journal is co-owned by BMJ to the co-owners of the Journal, to publish the Work in this journal and any other BMJ products and to exploit all rights, as set out in our licence.

The Submitting Author accepts and understands that any supply made under these terms is made by BMJ to the Submitting Author unless you are acting as an employee on behalf of your employer or a postgraduate student of an affiliated institution which is paying any applicable article publishing charge ("APC") for Open Access articles. Where the Submitting Author wishes to make the Work available on an Open Access basis (and intends to pay the relevant APC), the terms of reuse of such Open Access shall be governed by a Creative Commons licence – details of these licences and which Creative Commons licence will apply to this Work are set out in our licence referred to above.

Other than as permitted in any relevant BMJ Author's Self Archiving Policies, I confirm this Work has not been accepted for publication elsewhere, is not being considered for publication elsewhere and does not duplicate material already published. I confirm all authors consent to publication of this Work and authorise the granting of this licence.

For peer review only

Intermarriage and COVID-19 mortality among immigrants. A population-based cohort study from Sweden

Siddartha Aradhya[1], Maria Brandén[1,2], Sven Drefahl[1], Ognjen Obućina[3], Gunnar Andersson[1], Mikael Rostila[4,5], Eleonora Mussino[1], Sol P. Juárez[4,5*]

1. Stockholm University Demography Unit, Stockholm University, 106 91 Stockholm, Sweden

2. Institute for Analytical Sociology, Linköping University, 601 74 Norrköping, Sweden

3. Institut national d'études démographiques (INED), 9 cours des Humanités, 93322 Aubervilliers, France

4. Department of Public Health Sciences, Stockholm University, 106 91 Stockholm, Sweden

5. Centre for Health Equity Studies (CHESS), Stockholm University/Karolinska Institute, Stockholm, Sweden

[*] Corresponding author: Sol P. Juárez, sol.juarez@su.se Department of Public Health Sciences, Stockholm University, SE-106 91, Stockholm, Sweden

Keywords: COVID-19; immigrants; language barriers; mortality; Sweden

Word count. 3235

For peer review only - http://bmjopen.bmj.com/site/about/guidelines.xhtml

Intermarriage and COVID-19 mortality among immigrants. A population-based cohort study from Sweden

Abstract

Objectives. To evaluate the role of language proficiency and understanding of the healthcare system and health recommendations in explaining excess COVID-19 mortality among immigrants.

Design. Cohort study with follow-up between March 4, 2020 and February 23, 2021.

Setting. Swedish register-based study on all residents in Sweden.

Participants. 836,390 Stockholm residents in co-residential unions who were 30 years of age or older and alive on March 4th, 2020 and living in Sweden in December 2019.

Outcome measures. Cox regression models were conducted to assess the association between different constellations of immigrant-native couples (proxy for language proficiency and institutional awareness) and COVID-19 mortality and all other causes of deaths (2019 and 2020). Models were adjusted for relevant confounders.

Results. Compared to Swedish-Swedish couples (1.18 deaths per thousand person-years), both immigrants partnered with another immigrant and a native showed excess mortality for COVID-19 (HR 1.43; 95%CI 1.29, 1.58 and HR 1.24; 95%CI 1.10, 1.40, respectively) which translates to 1.37 and 1.28 deaths per thousand person-years. Moreover, similar results are found for natives partnered with an immigrant (HR 1.15; 95%CI 1.02, 1.29) which translates to 1.29 deaths per thousand person-years. Further analysis shows that immigrants from both high and low-middle income countries experience excess mortality also when partnered with a Swede. Moreover, patterns of mortality for all other causes of death show that immigrants, on average, have the same or lower mortality than natives regardless of the origin of their partner.

Conclusions. Factors related to language proficiency and institutional awareness are not likely explanations of the excess mortality from COVID-19 among immigrants.

Article Summary

- This study uses total population data with all deaths (from COVID-19 and other causes) in Sweden from March 4th 2020 to 23rd February 2021.
- We identified the origin of co-resident couples to evaluate the role of language proficiency and institutional awareness (e.g., healthcare system) in explaining excess COVID-19 mortality among immigrants.
- We compare COVID-19 mortality to all other causes of death during the pandemic and all-cause mortality one year prior to evaluate the relative impact of the pandemic within each group.
- The analyses do not include information on occupation, however the most vulnerable individuals are beyond retirement age.

For peer review only

For peer review only - http://bmjopen.bmj.com/site/about/guidelines.xhtml

International evidence has shown that immigrants and ethnic minorities are disproportionately at risk of severe COVID-19 complications and death[1–13]. In the context of an ongoing pandemic, an effort to understand the causes for why some groups are more affected is a public health priority.[14–17] In the case of immigrants, high-risk occupations, overcrowded accommodation, language barriers and access to healthcare, have been considered as potential explanations.[18] Recent studies, however, suggest that immigrants and minorities maintain an excess mortality even after controlling for socio-economic status and housing conditions.[3,19] Yet there is paucity of evidence on the role of language barriers and institutional awareness in explaining the COVID-19 excess mortality experienced by immigrants.

Sweden took a distinct approach to dealing with the COVID-19 pandemic as compared to other Western countries by not implementing lock downs or mask mandates and instead relied largely on recommendations. The authorities justified implementing a relatively less rigid approach by arguing that Swedes have a high level of trust in their institutions and as such follow governmental recommendations.[20,21] The strategy relied primarily on public health advice (regarding hygiene routines, social distancing and suspension from work, school or daycare in case of minor symptoms) *in lieu* of mandates which are not permitted under Swedish law.[20] The effectiveness of the adopted strategy strongly depends on the ability of all members of the society to understand the recommendations, which is a basic condition for their adherence. Under this rationale, it is unsurprising that excess mortality observed among immigrants[22]—especially concentrated among those with more distant origins—could be interpreted as a consequence of lower adherence to recommendations and/or related factors.

More specifically, it has been argued that immigrants may, as a result of inadequate language proficiency and institutional awareness, have a poor understanding of the healthcare system and of the recommendations during the COVID-19 pandemic putting them at higher risk of exposure and vulnerability to the virus. Analyzing intermarried immigrants present a unique opportunity to generate evidence for this explanation.

Within the field of immigrant integration, intermarriage has been considered the ultimate stage of acculturation[23–25] and is, both, a marker of and facilitator for integration. Marrying a native is strongly related to language abilities, knowledge of the host country's institutions and social practices, as well as the ethnic composition of one's social circle. As a result, intermarriage reflects the narrowing of socio-cultural distance between ethnic Swedes and immigrants, which renders it an ideal measure to evaluate the role of understanding and awareness of recommendations from Swedish authorities as explanations for the excess COVID-19 mortality among immigrants.

If understanding and awareness of recommendations (language *in primis*) explain the excess mortality, immigrants partnered with a Swede and, in particular, Swedes partnered with an immigrant should have similar mortality to Swedes partnered with a Swede. To this end, the aim of this study is to examine the association between socio-cultural integration and COVID-19 mortality by examining native-immigrant couple dyads—a well-regarded measure that has been shown to be a marker and facilitator of integration.

Methods

Study population

An observational cohort study was conducted using Swedish register data. The study includes all Swedish residents who were 30 years of age or older and were cohabiting with another adult who was at least 30 years of age and alive on March 4th, 2020, residing in Sweden in December 2019 (n= 4,019,418). This age restriction was established to ensure co-resident individuals were family members and not flat mates. The follow-up period was March 4, 2020 up until February 23, 2021. We excluded individuals who had not lived in Sweden in the two prior years (n=40,515), because they could not be linked to all records of data. In addition, we excluded individuals with missing data on country of birth (n=142) and income (n=15,405) of either partner. The final study population consists of 3,963,356 individuals (18.5% immigrants) (figure 1).

Patient and public involvement

No patient involved

For peer review only

For peer review only - http://bmjopen.bmj.com/site/about/guidelines.xhtml

Data

We use information from several Swedish administrative registers linked through personal identity numbers that are unique to each person with legal residence in Sweden. Data on deaths were retrieved from the Cause of Death Register. Socioeconomic and demographic variables (income, education, number of children, and region of residence) were drawn from the Longitudinal integrated database for health insurance and labor market studies (LISA), and residential information (type and crowdedness of the dwelling, and immigrant density in the neighborhood) were drawn from the Dwelling register. All covariates in our study are time-constant and either measured at the end of 2019 (all variables at the household and neighborhood level) or 2018 (highest education attained, sum of the individual net incomes of the two co-resident adults, total number of individuals in the household under 30). Information on age, sex, and country of birth stem from the Total Population Register. It is important to note that all individuals registered in Sweden are entitled to health care access.

Study variables

COVID-19 mortality was identified by the Swedish National Board of Health and Welfare (*Socialstyrelsen*), the agency responsible for the cause of death register. COVID-19 mortality was identified using the following ICD codes for the underlying cause of death: U07.1 (3,915 deaths), U07.2 (127 deaths), and B34.2 (2 deaths); for 522 more deaths ICD codes U07.1, U07.2 or B34.2 were listed as contributing causes of death, excluding mortality from all other causes of death (30,374 deaths). Given the timeliness of the data, the assignment of the underlying cause of death should be understood as preliminary.

Immigrant-native couple types were created by combining information from the dwelling and the total population register to create the couple type which include two individuals of at least 30 years of age co-residing in the same household. The variable is classified into the following four ego-partner categories using information on country of birth: (i) native-native, (ii) native-immigrant, (iii) immigrant-native, and (iv) immigrant-immigrant. We chose (i) native-native couples as the reference group for our analyses as they a) constitute the largest category among all groups considered and b) represent the

For peer review only - http://bmjopen.bmj.com/site/about/guidelines.xhtml

institutional awareness and language of the host population from which we expect other groups to deviate. We further disaggregated the groups by immigrant's origins defined according to the World Bank classification based on the Gross National Incomes (GNI) per capita using the WB Atlas method[26] as low-middle and high income countries.

We derived individual income and calculated the sum of the two partners' net incomes (household), categorized into tertiles based on all adult residents of Sweden. We derived education data from Swedish educational registers and categorize our population into four categories; those with primary schooling, secondary schooling, post-secondary education, and those with missing information on education. Missing information on education is generally very low but 88% of those with missing education are immigrants. We additionally performed multiple imputation to test how the missing values for education impact our results (Supplementary Figure 1 and Supplementary Table 1). From the Swedish Dwelling register we accessed information on size of the dwelling and a unique dwelling code which enables us to link individuals who live together in a household and determine co-residence. From this information we create: the number of individuals per square meter in the household (with a separate category for a small group of individuals, due to missing information on square meters in some detached houses), the number of individuals living in the household under the age of 30, and dwelling type (multi-family, single family, or care home). We include in our model also the share of immigrants in the local neighborhood, DeSO (a smaller subdivision of administrative areas based on demographic characteristics produced by the Swedish administrative statistics).

Statistical analysis

We conducted Cox proportional hazards regressions (using age as the timescale) to estimate Hazard Ratios (HR) and 95% Confidence Intervals (CI) for the association between immigrant-native couple type and COVID-19 mortality. Individuals exited the study by (1) dying between March 5, 2020 and February 23, 2021, or (2) being alive on February 23, 2021. We estimated two separate regressions estimating the cause-specific hazard of dying from COVID-19, right-censoring all individuals that die from other causes and (2) the cause-specific hazard of dying from other causes than COVID-19, right-

For peer review only - http://bmjopen.bmj.com/site/about/guidelines.xhtml

censoring all individuals that die from COVID-19. In addition, we conducted Cox regressions for dying from all causes of death that occurred between March 5, 2019 and February 23, 2020 (31,653 deaths)—the same period we observe COVID-19 deaths twelve contiguous months prior to the start of the pandemic. Since mortality from COVID-19 and other causes of deaths during the study window are not fully independent of each other, our estimates for all-cause mortality between March 5, 2019 and February 23, 2020 were used to evaluate the robustness of our estimates for mortality during the pandemic. In addition, the comparison between all-cause mortality one year prior and mortality from causes other than COVID-19 during the pandemic, by immigrant-native couple type, will allow us to examine whether the latter has a distinctive role in relation to the pandemic.

Two models were estimated: 1) a simple model with age as the time scale adjusted for sex and 2) the same model with further adjustments. In the latter analysis we adjust for education, neighborhood immigrant density, and region of residence fixed-effects that are confounders, as well as factors that are on the causal pathway that have been previously used to explain the excess mortality of immigrants (i.e, number of individuals in the household below the age of 30, dwelling type, square meters per person in the dwelling, household income). In addition, we conducted two sensitivity analyses. One in which we examined the partner's origin among Swedish-migrant partnerships (high or low-middle income country) to check whether patterns are consistent across groups (Supplementary Figure 2) and, a second, in which we excluded all individuals born in Sweden with at least one foreign-born parent (Supplementary Figure 3). Both sensitivity analyses rendered similar results. All analyses were conducted using Stata Statistical Software: Release 16 (StataCorp LP, College Station, Texas).

This study was approved by the Central Ethical Review Board in 2020 (Dnr 2020-02199).

Results

During the 3,759,610 person-years of observation, 4,564 COVID-19 deaths occurred in our study population between March 5, 2020 and February 23, 2021. Table 1 shows the distribution of population at risk and deaths by all covariates. In our population, 6.09% of

For peer review only - http://bmjopen.bmj.com/site/about/guidelines.xhtml

individuals are in immigrant-native couples. Native-native couples show the lowest deaths per thousand person-years (1.18/1000), whereas all other couple types show higher death rates of COVID-19 mortality (approximately 1.3-1.4 per thousand person-years). Of the study population, 6.55% of COVID-19 deaths were attributed to native-immigrant mixed couples and 14.04% to immigrant-immigrant couples.

Figure 2 displays mortality risks from COVID-19, all other causes of death during the pandemic, and all-causes of death one year prior across couple types with native-native as the reference (see Supplementary Table 2 for estimates). Panel A presents models adjusted for age and sex and panel B presents the estimates including adjustments. In A, individuals in immigrant-immigrant couples show the highest HR of dying from COVID-19 (HR 2.47; 95%CI: 2.27, 2.69) and those in native-native couples the lowest (reference group) while natives (HR 1.40; 95%CI: 1.25, 1.58) and immigrants (HR 1.50; 95%CI: 1.33, 1.69) in mixed couples showed intermediate mortality levels. All-causes of death in the year prior to the pandemic and all-other causes of death during the pandemic show little differences between couple constellations. After adjustments (panel B), differences across groups attenuate, but the gradient in COVID-19 mortality remains. Individuals in immigrant-immigrant couples still display the highest HR of dying from COVID-19 relative to the reference group (HR 1.43; 95%CI: 1.29, 1.58), followed by those in immigrant-native couples (HR 1.24; 95%CI: 1.10, 1.40) and native-immigrant couples (HR 1.15; 95%CI: 1.02, 1.29). It is important to note that there is no statistically significant difference in the risk of COVID-19 mortality between immigrant-native and immigrant-immigrant couples. An opposite gradient is observed with respect to all-cause mortality in the year prior to the pandemic and all other causes of death during the pandemic, with immigrant-immigrant couples displaying the lowest mortality.

Figure 3 is an extension of figure 2 disaggregating the immigrant population by income level of their country of birth (see Supplementary Table 3 for estimates). In panel A, there is elevated HRs across all origin groups relative to native-native couples. Immigrants in LMIC-immigrant couples display the highest HR relative to the reference group (HR 3.60; 95%CI 3.25, 3.99) followed by those in LMIC-native couples (HR 1.91; 95%CI 1.42, 2.57).

For peer review only - http://bmjopen.bmj.com/site/about/guidelines.xhtml

Natives in native-immigrant couples, where HIC & LMIC have been pooled, (HR 1.41; 95%CI 1.25, 1.58), HIC-native (HR 1.44; 95%CI 1.27, 1.64), and immigrants in HIC-immigrant (HR 1.54; 95%CI 1.34, 1.77) couples display relatively similar HRs. After adjustment (panel B), all groups with an immigrant still display higher HRs relative to native-native couples, but at a lower level. LMIC-immigrant couples experience a particularly strong reduction in their HRs (HR 1.84; 95%CI 1.62, 2.09). Compared to LMIC-native couples, their HR remains slightly higher (HR 1.35; 95%CI 1.00, 1.82). We also find that HIC-native (HR 1.23; 95%CI 1.08, 1.40) and HIC-immigrant (HR 1.11; 95%CI 0.97, 1.28) couples display higher HRs relative to the reference group.

Sensitivity analyses showed that disaggregating the origin of immigrants in native-immigrant partnerships (Supplementary Figure 2) and excluding second-generation Swedes from the Swedish-born population (Supplementary Figure 3) produce no further differentials in mortality risks.

Discussion

Our study shows that immigrants have excess COVID-19 mortality regardless of the origin of their partner, where having a Swedish-born partner is only partially protective against COVID-19 mortality for immigrants. These findings challenge hypotheses that factors related to language proficiency and institutional awareness are relevant factors when explaining the excess mortality from COVID-19 among immigrants.

Language has been considered a vital component of integration and relevant for accessing information on other types of medical treatments and health outcomes[27]. Although we cannot test this aspect directly, our study shows that lack of language barriers do not seem to be entirely protective for those with a native speaker in the household (immigrants partnered with a Swede), which raises the question as to whether language barriers may at all explain the excess mortality among immigrants partnered with another immigrant. Given that the COVID-19 pandemic is a unique occurrence that was accompanied by global diffusion of information, one can argue that even those with presumably little Swedish proficiency and without previous knowledge of the Swedish

For peer review only - http://bmjopen.bmj.com/site/about/guidelines.xhtml

system (immigrants cohabiting with another immigrant) have been exposed to recommendations offered in their native languages from either public health officials from their countries of origin or via other international channels. In fact, the information that they may have received from international sources may be more relevant for specific immigrant populations, for example, how to best protect oneself when observing cultural or religious practice. Prior studies in clinical settings have shown that culturally adapted information is associated with better health access and outcomes.[27]

Beyond allowing us to disentangle the role of language barriers and lack of understanding of the healthcare system and recommendations in explaining the excess COVID-19 mortality among immigrants, our study provides suggestive evidence that differential exposure to the virus and not susceptibility is the culprit. [28] Although it is true that Swedes partnered with a Swede show the lowest mortality, those partnered with an immigrant experience higher COVID-19 mortality. Given that Swedes do not experience language barriers or lack institutional awareness and that biological susceptibility cannot be transmitted between partners, Swedes partnered with immigrants are likely to be at either higher exposure to the virus or impacted by the social susceptibility of their partner. Swedes in mixed partnerships may be exposed to similar social environments and/or risk factors as immigrants that place them at a higher risk of exposure as compared to Swedes partnered with another Swede. For example, they may have more transnational contacts or are impacted by some of the same social risk factors as immigrants. Moreover, the higher mortality experienced by natives partnered with immigrants could be related to the disadvantages faced by the immigrant partner either via discrimination or a higher exposure to the pandemic (e.g., having a frontline or precarious occupation). Albeit this type of exposure in mixed partnerships might not be at the same level as for immigrants partnered with another immigrant (as our results with a gradient in mortality suggests).

In this study, immigrants in different family constellations show higher levels of COVID-19 deaths than the majority of natives, after adjustment for a wide range of individual- and contextual-level factors, including education, income, housing conditions, and neighborhood immigrant density. This set of adjustments also partly accounts for a

number of socially patterned chronic health conditions and comorbidities, e.g., insulin resistance, hypertension, smoking and obesity, which have been suggested as risk factors for severe cases of COVID-19.[17]

This study has a number of contributions and strengths, First, this is the only study to date to examine socio-cultural integration as the mechanism behind the disproportionate burden that COVID-19 has placed on immigrant communities, by comparing the mortality of native-native, immigrant-native, and immigrant-immigrant couples in Sweden. A major strength of our study is that we have complete coverage of the total population and all deaths in Sweden from the start of the pandemic until February 2021 for both COVID-19 and remaining causes of death. Thus, our analysis does not suffer from selection into our study population. We have similar high-quality data for the year prior to the pandemic, which allows for an unbiased comparison of mortality patterns between the two years. The comparison between couple types with respect to mortality from all other causes during the pandemic and all-cause mortality in the year prior strengthens our findings as it demonstrates that the excess mortality from COVID-19 is observed in couples that show no excess mortality from other causes before and during the pandemic.

Despite its strengths, this study has also some limitations that are worth mentioning. Although the Swedish population registers hold high quality and have many advantages, they capture de jure rather than de facto characteristics of individuals. With respect to our measure of partnership, 82% of the couples in our data are either married or have shared children while the remaining 18% co-reside unmarried without common children. However, non-marital cohabitation is very common in Sweden, while flat-sharing is not[29], and particularly among individuals above their 30s. It is therefore fair to assume that a substantive share in this remaining group is indeed cohabiting in an amorous relationship, a group that is often overlooked in international studies of health and mortality because of the lack of data. A final limitation is that we do not include of information on occupation. Specifically, it has been hypothesized that immigrants are more likely to work in 'front-line' occupations; however, recent research in Sweden has shown that occupational exposure is not a risk factor for COVID-19 mortality. Furthermore, it is worth noting that a

For peer review only - http://bmjopen.bmj.com/site/about/guidelines.xhtml

majority of deaths are occurring among retired individuals with no attachment to the labor market.

In conclusion, our study shows that being partnered with a native does not close the gap in COVID-19 mortality with natives even after adjusting for a wide range of possible confounders on both individual- couple- and residential level. As such, these findings show that lack of awareness of the Swedish recommendations and language barriers do not explain the excess COVID-19 mortality of immigrants. At the same time, however, Swedes partnered with immigrants also show equal excess mortality compared to Swedish couples, suggesting that these groups experience higher COVID-19 mortality due to higher exposure to the virus.

For peer review only

Ethical approval: The analyses have been approved by the Swedish ethical-vetting authority, Dnr 2020-02199.

Contributorship statement: SA, MB and SJ conceived the study and were responsible for the planning. GA and MB provided the data, MB analyzed the data, SA, SJ, SD, EM, MB designed the analysis with contributions from OO, MR, GA. SA, SJ, SD, EM, MB, OO, MR, GA contributed to the interpretation of the data. SA and SJ drafted the manuscript with substantive contributions from EM, SD, MB, OO, MR, GA. All authors approved the final version of the manuscript.

Data sharing: This study is produced under the Swedish Statistics Act, where privacy concerns restrict the availability of register data for research. Aggregated data can be made available by the authors, conditional on ethical vetting. The authors access the individual-level data through Statistics Sweden's micro-online access system MONA.

Competing Interest statement: No, there are no competing interests for any author

Acknowledgements: We thank Thomas Niedomysl of Region Halland, Petra Westin of the National Board of Health and Welfare, and Simon Kurt of Statistics Sweden for their invaluable role in providing the data.

Funding: The Swedish Research Council for Health, Working Life and Welfare (FORTE), grant numbers 2016-07115, 2016-07105, 2016-07128, 2019-00603, The Swedish Research Council (VR), grant number 2018-01825 and The Swedish Foundation for Humanities and Social Sciences (Riksbankens Jubileumsfond), grant M18-0214:1.

For peer review only - http://bmjopen.bmj.com/site/about/guidelines.xhtml

References:

1. Pan, D. *et al.* The impact of ethnicity on clinical outcomes in COVID-19: A systematic review. *EClinicalMedicine* **23**, 100404 (2020).
2. Hayward, S. E. *et al.* Clinical outcomes and risk factors for COVID-19 among migrant populations in high-income countries: A systematic review. *Journal of Migration and Health* **3**, 100041 (2021).
3. Drefahl, S. *et al.* A population-based cohort study of socio-demographic risk factors for COVID-19 deaths in Sweden. *Nat Commun* **11**, 5097 (2020).
4. Aldridge, R. W. *et al.* Black, Asian and Minority Ethnic groups in England are at increased risk of death from COVID-19: indirect standardisation of NHS mortality data. *Wellcome Open Res* **5**, 88 (2020).
5. Rostila, M. *et al.* Disparities in COVID-19 deaths by country of birth in Stockholm, Sweden: A total population based cohort study. *American Journal of Epidemiology* (2021) doi:10.1093/aje/kwab057.
6. Niedzwiedz, C. L. *et al.* Ethnic and socioeconomic differences in SARS-CoV-2 infection: prospective cohort study using UK Biobank. *BMC Med* **18**, 160 (2020).
7. Ross, J., Diaz, C. M. & Starrels, J. L. The Disproportionate Burden of COVID-19 for Immigrants in the Bronx, New York. *JAMA Intern Med* (2020) doi:10.1001/jamainternmed.2020.2131.
8. Millett, G. A. *et al.* Assessing Differential Impacts of COVID-19 on Black Communities. *Annals of Epidemiology* S1047279720301769 (2020) doi:10.1016/j.annepidem.2020.05.003.
9. Williamson, E. J. *et al.* Factors associated with COVID-19-related death using OpenSAFELY. *Nature* **584**, 430–436 (2020).

For peer review only - http://bmjopen.bmj.com/site/about/guidelines.xhtml

10. Indseth, T. *et al.* COVID-19 among immigrants in Norway, notified infections, related hospitalizations and associated mortality: A register-based study. *Scand J Public Health* **49**, 48–56 (2021).

11. Raharja, A., Tamara, A. & Kok, L. T. Association Between Ethnicity and Severe COVID-19 Disease: a Systematic Review and Meta-analysis. *J. Racial and Ethnic Health Disparities* (2020) doi:10.1007/s40615-020-00921-5.

12. Staetsky, L. D. Elevated Jewish Mortality from Coronavirus in England and Wales: An Epidemiological and Demographic Detective Story. *Cont Jewry* (2021) doi:10.1007/s12397-021-09366-2.

13. Vanthomme, K., Gadeyne, S., Lusyne, P. & Vandenheede, H. A population-based study on mortality among Belgian immigrants during the first COVID-19 wave in Belgium. Can demographic and socioeconomic indicators explain differential mortality? *SSM - Population Health* **14**, 100797 (2021).

14. Statement by the EUPHA Migrant and ethnic minority health section on COVID-19 – CALL FOR ACTION: Reducing the impact of the coronavirus crisis on those 'left behind' – Disadvantaged Migrants and Ethnic Minorities (MEM). (2020).

15. Pareek, M. *et al.* Ethnicity and COVID-19: an urgent public health research priority. *The Lancet* **395**, 1421–1422 (2020).

16. Bhala, N., Curry, G., Martineau, A. R., Agyemang, C. & Bhopal, R. Sharpening the global focus on ethnicity and race in the time of COVID-19. *The Lancet* **0**, (2020).

17. Khunti, K., Singh, A. K., Pareek, M. & Hanif, W. Is ethnicity linked to incidence or outcomes of covid-19? *BMJ* **369**, (2020).

For peer review only

For peer review only - http://bmjopen.bmj.com/site/about/guidelines.xhtml

18. Diaz, E. *et al. Situational brief: Migration and Covid-19 in Scandinavian countries*. https://www.migrationandhealth.org/migration-covid19-briefs (2020).

19. Rostila, M. *et al.* Disparities in covid-19 deaths by country of birth in Stockholm, Sweden: A total population based cohort study. *Stockholm Research Reports in Demography. Preprint.* (2020) doi:https://doi.org/10.17045/sthlmuni.12852854.v1.

20. Pierre, J. Nudges against pandemics: Sweden's COVID-19 containment strategy in perspective. *Policy and Society* **39**, 478–493 (2020).

21. Paterlini, M. 'Closing borders is ridiculous': the epidemiologist behind Sweden's controversial coronavirus strategy. *Nature* **580**, 574–574 (2020).

22. Folkhälsomydigheten. *Veckorapport om covid-19, vecka 15*. https://www.folkhalsomyndigheten.se/globalassets/statistik-uppfoljning/smittsamma-sjukdomar/veckorapporter-covid-19/2020/covid-19-veckorapport-vecka-15.pdf (2020).

23. Alba, R. D. & Golden, R. M. Patterns of Ethnic Marriage in the United States. *Social Forces* **65**, 202–223 (1986).

24. Lieberson, S. & Waters, M. C. Ethnic groups in flux: the changing ethnic responses of American whites. *The Annals of the American Academy of Political and Social Science* **487**, 79–91 (1986).

25. Kalmijn, M. Intermarriage and Homogamy: Causes, Patterns, Trends. *Annual Review of Sociology* **24**, 395–421 (1998).

26. The World Bank. GNI, Atlas method (current US$) | Data. *DataBank* https://data.worldbank.org/indicator/NY.GNP.ATLS.CD.

For peer review only

For peer review only - http://bmjopen.bmj.com/site/about/guidelines.xhtml

27. Flores, G. The Impact of Medical Interpreter Services on the Quality of Health Care: A Systematic Review. *Med Care Res Rev* **62**, 255–299 (2005).

28. Katikireddi, S. V. *et al.* Unequal impact of the COVID-19 crisis on minority ethnic groups: a framework for understanding and addressing inequalities. *J Epidemiol Community Health* jech-2020-216061 (2021) doi:10.1136/jech-2020-216061.

29. SCB. *Ensam, med partner eller kompisboende Vad säger hushållsregistret?* https://www.scb.se/contentassets/cfe7690018d741798939bd8a6d087219/be0701_2015i2018_br_be51br1901.pdf (2019).

For peer review only

Table 1. Description of the study population, number, proportion of deaths and death rates

	Total		COVID-19		Other cause of death		Exposure time in years	COVID-19 deaths per 1000 years
	N at March 5, 2020	%	Deaths March 5, 2020 – February 23, 2021	%	Deaths March 5, 2020 – February 23, 2021	%		
**Couple type**								
Native-native	2,984,648	75.31	3,330	72.96	24,846	81.80	2,830,382	1.18
Native-immigrant	245,124	6.18	299	6.55	1,739	5.73	232,563	1.29
Immigrant-native	241,185	6.09	294	6.44	1,507	4.96	228,949	1.28
Immigrant-immigrant	492,399	12.42	641	14.04	2,282	7.51	467,716	1.37
**Couple type, detailed**								
Native-native	2,984,648	75.31	3,330	72.96	24,846	81.80	2,830,382	1.18
Native-immigrant	245,124	6.18	299	6.55	1,739	5.73	232,563	1.29
HIC-native	140,281	3.54	249	5.46	1,294	4.26	132,929	1.87
HIC-immigrant	121,148	3.06	215	4.71	1,170	3.85	114,765	1.87
LMIC-native	100,904	2.55	45	0.99	213	0.70	96,020	0.47
LMIC-immigrant	371,251	9.37	426	9.33	1,112	3.66	352,951	1.21
**Sex**								
Male	1,992,097	50.26	3,145	68,91	19,003	62.56	1,887,523	1.67
Woman	1,971,259	49.74	1,419	31.09	11,371	37.44	1,872,087	0.76
**Education**								
Primary	589,969	14.89	1,810	39.66	11,495	37.84	555,860	3.26
Secondary	1,687,832	42.59	1,732	37.95	12,069	39.73	1,601,553	1.08
Post-secondary	1,651,905	41.68	900	19.72	6,420	21.14	1,570,393	0.57
Missing	33,650	0.85	122	2.67	390	1.28	31,804	3.84
**Household income (tertile)**								
Lowest	1,289,265	32.53	3,544	77.65	22,358	73.61	1,216,124	2.91
Middle	1,336,367	33.72	625	13.69	5,008	16.49	1,270,568	0.49
Highest	1,337,724	33.75	395	8.65	3,008	9.90	1,272,918	0.31
**Housing type**								
Multi-family	1,283,364	32.38	2,248	49.26	11,797	38.84	1,216,054	1.85
Single-family	2,662,463	67.18	1,930	42.29	16,946	55.79	2,527,860	0.76
Care home	17,529	0.44	386	8.46	1,631	5.37	15,696	24.59
**Number of people under 30 in the household**								
0	2,079,106	52.46	4,244	92.99	28,211	92.88	1,965,558	2.16
1	614,976	15.52	180	3.94	1,191	3.92	585,276	0.31
2	870,872	21.97	85	1.86	647	2.13	829,379	0.10
3+	398,402	10.05	55	1.21	325	1.07	379,397	0.14
**m2/person in the household (crowdedness)**								
0-	325,441	8.21	325	7.12	1,463	4.82	309,194	1.05
20-	814,282	20.55	464	10.17	2,809	9.25	774,226	0.60
30-	913,084	23.04	1,240	27.17	7,232	23.81	865,941	1.43
40-	1,127,740	28.45	1,662	36.42	11,264	37.08	1,068,381	1.56
60-	750,691	18.94	861	18.87	7,468	24.59	711,339	1.21

For peer review only

For peer review only - http://bmjopen.bmj.com/site/about/guidelines.xhtml

Missing	32,118	0.81	12	0.26	138	0.45	30,530	0.39
**Share immigrants in DeSO (%)**								
0-	1,306,913	32.97	958	20.99	9,358	31.40	1,240,219	0.77
0.10-	943,691	23.81	972	21.30	7,318	24.09	895,256	1.09
0.15-	659,303	16.63	858	18.80	5,003	16.47	625,400	1.37
0.20-	556,222	14.03	765	16.76	4,575	15.06	527,391	1.45
0.30-	349,234	8.81	637	13.96	2,896	9.53	331,065	1.92
0.50-	147,993	3.73	374	8.19	1,044	3.44	140,278	2.67
**TOTAL**	3,963,356	100.0	4,564	100.0	30,374	100.0	3,759,610	1.21

Figure legends.

Figure 1. Selection flow and final sample

Figure 2. Hazard Ratios for the risk of dying from COVID-19, other causes of death during the pandemic, and all-cause mortality in the year prior to the pandemic by couple dyad. Model A is adjusted for age and sex only and model B includes full adjustment (reference group: native-native couples)

Figure 3. Hazard Ratios for the risk of dying from COVID-19, other causes of death during the pandemic, and all-cause mortality in the year prior to the pandemic by couple dyad disaggregated by LMIC and HIC immigrants. Model A is adjusted for age and sex only and model B includes full adjustment (reference group: native-native couples)

For peer review only - http://bmjopen.bmj.com/site/about/guidelines.xhtml

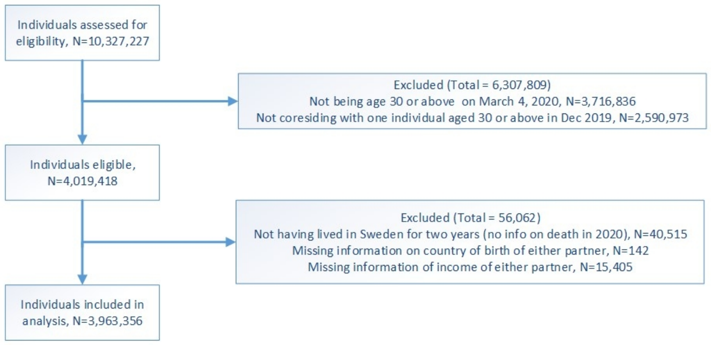

Figure 1

338x190mm (96 x 96 DPI)

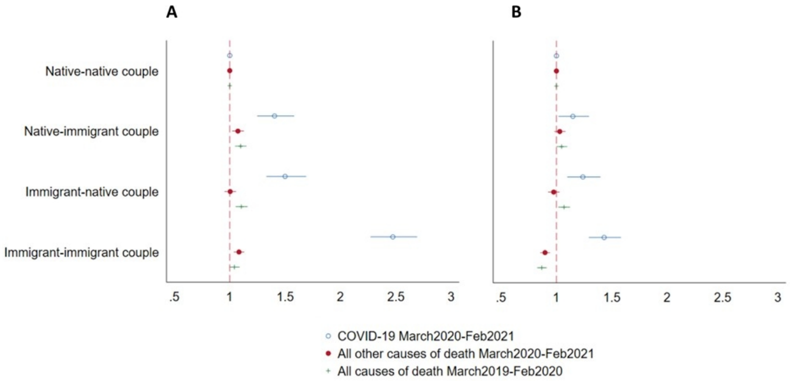

Figure 2

338x190mm (96 x 96 DPI)

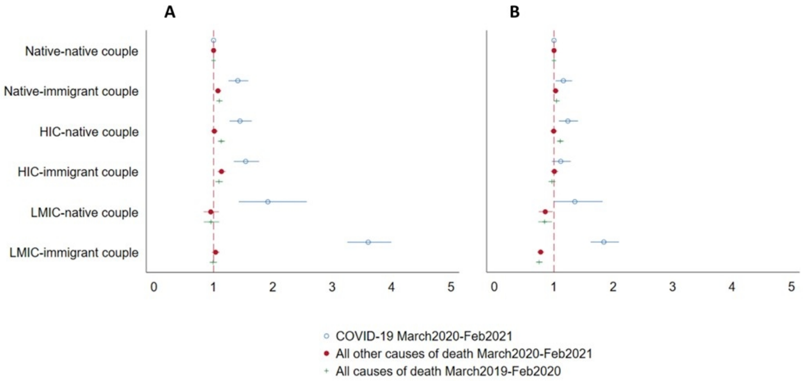

Figure 3

338x190mm (96 x 96 DPI)

Supplementary table 1: Descriptive table displaying distributions of all covariables with and without missing in education and dwelling size.

	Study population (with missing education and dwelling size)	Study population (without missing education)	Study population (without missing dwelling size)
**Couple dyads**			
Native-native	75.31	75.86	75.25
Native-immigrant	6.18	6.21	6.19
Immigrant-native	6.09	5.99	6.09
Immigrant-immigrant	12.42	11.94	12.47
**Sex**			
Man	50.26	50.21	50.26
Woman	49.74	49.79	49.74
**Education**			
Primary	14.89	15.01	14.91
Secondary	42.59	42.95	42.58
Post-secondary	41.68	42.04	41.66
Missing	0.85		0.85
**Income HH disposable Inc (tertlies)**			
1	32.53	32.22	32.58
2	33.72	33.85	33.73
3	33.75	33.93	33.69
**Housing type**			
Multi-family dwelling	32.38	32.11	32.64
Single-family dwelling	67.18	67.47	66.91
Care home	0.44	0.42	0.45
**# of individuals in the household**			
2	52.46	52.47	52.50
3	15.52	15.50	15.53
4	21.97	22.02	21.93
5+	10.05	10.01	10.04
**Sqm per person**			
0	8.21	8.08	8.28
-20	20.55	20.50	20.71
-30	23.04	23.04	23.23
-40	28.45	28.54	28.69
-60	18.94	19.03	19.10
Missing	0.81	0.81	
**Immigrants in DeSO**			
0	32.97	33.15	32.77
.1-	23.81	23.88	23.82
.15-	16.63	16.64	16.70
.2-	14.03	14.01	14.09
.3-	8.81	8.72	8.86
.5-	3.73	3.61	3.76
**Region of residence**			
Stockholms län	21.11	21.01	21.13

For peer review only

Uppsala län	3.57	3.57	3.57
Södermanlands län	2.91	2.92	2.90
Östergötlands län	4.64	4.65	4.65
Jönköpings län	3.73	3.74	3.73
Kronobergs län	2.05	2.04	2.05
Kalmar län	2.60	2.60	2.60
Gotlands län	0.59	0.60	0.59
Blekinge län	1.68	1.68	1.67
Skåne län	13.24	13.20	13.24
Hallands län	3.59	3.60	3.59
Västra Götalands län	16.71	16.73	16.75
Värmlands län	2.89	2.88	2.89
Örebro län	2.95	2.96	2.95
Västmanlands län	2.74	2.74	2.75
Dalarnas län	2.95	2.96	2.93
Gävleborgs län	2.89	2.91	2.90
Västernorrlands län	2.56	2.57	2.56
Jämtlands län	1.29	1.30	1.28
Västerbottens län	2.75	2.76	2.73
Norrbottens län	2.56	2.57	2.54

For peer review only

Supplementary table 2: Regression results for the risk of dying from COVID-19, other causes of death during the pandemic, and all-cause mortality in the year prior to the pandemic by couple dyad

	Model 1: Figure 2A						Model 2: Figure 2B					
	COVID- 19		Other COD Mar 5, 2020- Feb 23, 2021		All COD Mar 5, 2019 – Feb 23, 2020		COVID- 19		Other COD Mar 5, 2020- Feb 23, 2021		All COD Mar 5, 2019 – Feb 23, 2020	
	HR	95% CI	HR	95% CI	HR	95% CI	HR	95% CI	HR	95% CI	HR	95% CI
**Partnership type**												
Native-native	1.00	1.00	1.00	1.00	1.00	1.00	1.00	1.00	1.00	1.00	1.00	1.00
Native-immigrant	1.40	(1.25, 1.58)	1.07	(1.02, 1.13)	1.10	(1.05, 1.15)	1.15	(1.02, 1.29)	1.03	(0.98, 1.08)	1.05	(1.00, 1.10)
Immigrant-native	1.50	(1.33, 1.69)	1.00	(0.95, 1.06)	1.11	(1.05, 1.16)	1.24	(1.10, 1.40)	0.98	(0.93, 1.03)	1.07	(1.02, 1.12)
Immigrant-immigrant	2.47	(2.27, 2.69)	1.08	(1.04, 1.13)	1.04	(1.00, 1.09)	1.43	(1.29, 1.58)	0.90	(0.85, 0.94)	0.87	(0.83, 0.91)
Years under risk	3,759,610		3,759,610		3,764,370		3,759,610		3,759,610		3,764,370	
N events	4564		30,374		31,653		4564		30,374		31,653	
N	3,963,356		3,963,356		3,966,345		3,963,356		3,963,356		3,966,345	

Model 1 includes adjustments for sex; Model 2 includes adjustments for sex, household income, education, housing type, number of individuals in the household, m^2/person in the household (crowdedness), share of immigrants in the neighborhood of residence, and region of residence

For peer review only

Supplementary table 3: Regression results for the risk of dying from COVID-19, other causes of death during the pandemic, and all-cause mortality in the year prior to the pandemic by couple dyad disaggregated by LMIC and HIC immigrants.

	Model 3: Figure 3A						Model 4: Figure 3B					
	COVID- 19		Other COD Mar 5, 2020- Feb 23, 2021		All COD Mar 5, 2019 – Feb 23, 2020		COVID- 19		Other COD Mar 5, 2020- Feb 23, 2021		All COD Mar 5, 2019 – Feb 23, 2020	
	HR	95% CI	HR	95% CI	HR	95% CI	HR	95% CI	HR	95% CI	HR	95% CI
**Partnership type**												
Native-native	1.00	1.00	1.00	1.00	1.00	1.00	1.00	1.00	1.00	1.00	1.00	1.00
Native-immigrant	1.41	(1.25, 1.58)	1.07	(1.02, 1.13)	1.10	(1.05, 1.15)	1.16	(1.03, 1.30)	1.03	(0.98, 1.08)	1.04	(0.99, 1.09)
HIC-native	1.44	(1.27, 1.64)	1.01	(0.96, 1.07)	1.13	(1.07, 1.19)	1.23	(1.08, 1.40)	0.99	(0.94, 1.05)	1.11	(1.05, 1.16)
HIC-immigrant	1.54	(1.34, 1.77)	1.13	(1.07, 1.20)	1.09	(1.03, 1.16)	1.11	(0.97, 1.28)	1.01	(0.95, 1.07)	0.97	(0.91, 1.02)
LMIC-native	1.91	(1.42, 2.57)	0.95	(0.83, 1.09)	0.96	(0.84, 1.09)	1.35	(1.00, 1.82)	0.85	(0.74, 0.98)	0.84	(0.74, 0.97)
LMIC-immigrant	3.60	(3.25, 3.99)	1.03	(0.97, 1.10)	0.99	(0.93, 1.06)	1.84	(1.62, 2.09)	0.77	(0.72, 0.83)	0.75	(0.70, 0.81)
Years under risk	3,759,610		3,759,610		3,764,370		3,759,610		3,759,610		3,764,370	
N events	4564		30,374		31,653		4564		30,374		31,653	
N	3,963,356		3,963,356		3,966,345		3,963,356		3,963,356		3,966,345	

For peer review only

Model 3 includes adjustments for sex; Model 4 includes adjustments for sex, household income, education, housing type, number of individuals in the household, m^2/person in the household (crowdedness), share of immigrants in the neighborhood of residence, and region of residence

For peer review only

Supplementary figure 1: Hazard Ratios for the risk of dying from COVID-19 by couple dyad, including estimates from sensitivity analyses using multiple imputations to adjust for missing education and only complete cases

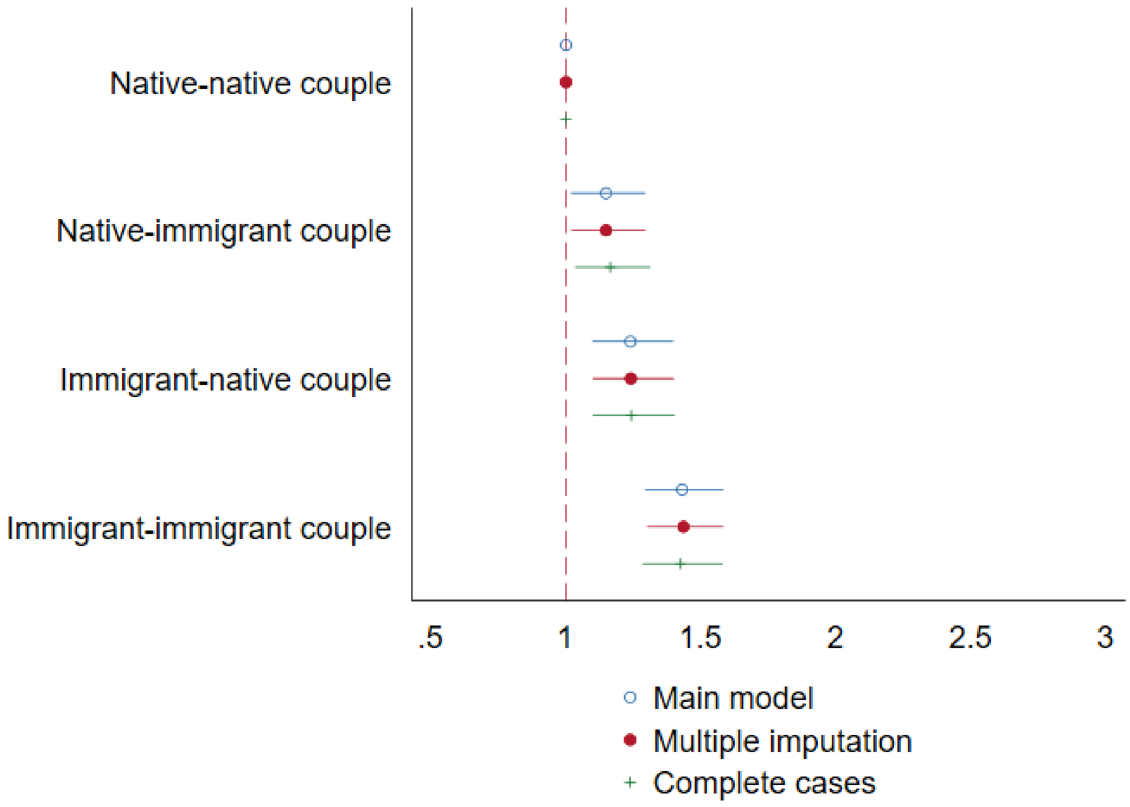

Supplementary figure 2: Hazard Ratios for the risk of dying from COVID-19, other causes of death during the pandemic, disaggregating native-immigrant couples by partner origin. Model includes full adjustment (reference group: native-native couples) (N=3,229,772)

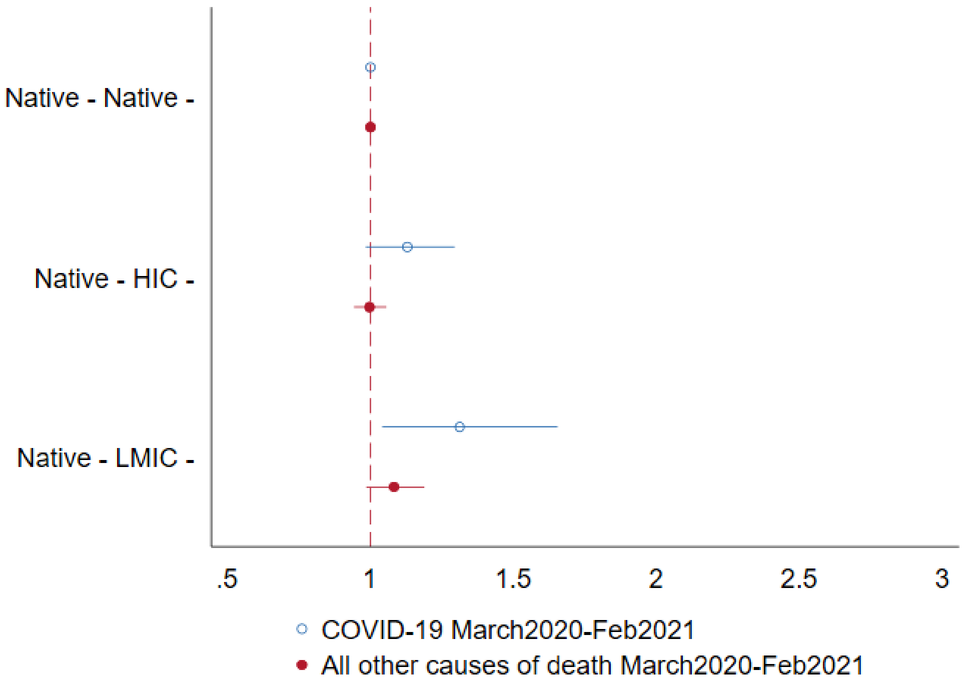

review only

Supplementary figure 3: Hazard Ratios for the risk of dying from COVID-19 by couple dyads excluding second generation immigrants. Model includes full adjustment (reference group: native-native couples) (N= 3,593,764)

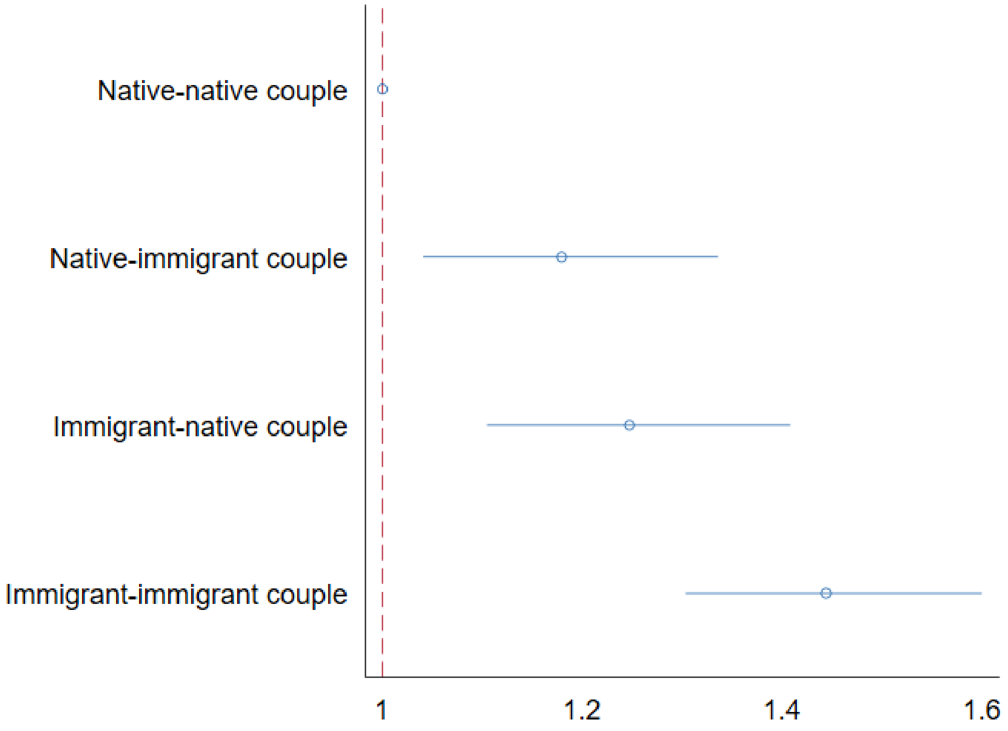

For peer review only

For peer review only

STROBE Statement—checklist of items that should be included in reports of observational studies

	Item No	Recommendation
**Title and abstract**	1	(*a*) Indicate the study's design with a commonly used term in the title or the abstract **Yes**
		(*b*) Provide in the abstract an informative and balanced summary of what was done and what was found **Yes**
**Introduction**		
Background/rationale	2	Explain the scientific background and rationale for the investigation being reported **Page 5**
Objectives	3	State specific objectives, including any prespecified hypotheses **Page 5-6**
**Methods**		
Study design	4	Present key elements of study design early in the paper **Pages 6**
Setting	5	Describe the setting, locations, and relevant dates, including periods of recruitment, exposure, follow-up, and data collection **Page 6**
Participants	6	*Cohort study*—Give the eligibility criteria, and the sources and methods of selection of participants. Describe methods of follow-up **Pages 6 and page 8** *Case-control study*—Give the eligibility criteria, and the sources and methods of case ascertainment and control selection. Give the rationale for the choice of cases and controls *Cross-sectional study*—Give the eligibility criteria, and the sources and methods of selection of participants
		(*b*) *Cohort study*—For matched studies, give matching criteria and number of exposed and unexposed *Case-control study*—For matched studies, give matching criteria and the number of controls per case
Variables	7	Clearly define all outcomes, exposures, predictors, potential confounders, and effect modifiers. Give diagnostic criteria, if applicable **Pages 6-7**
Data sources/ measurement	8*	For each variable of interest, give sources of data and details of methods of assessment (measurement). Describe comparability of assessment methods if there is more than one group **Pages 6-7**
Bias	9	Describe any efforts to address potential sources of bias **Pages 8 and 9**
Study size	10	Explain how the study size was arrived at **Page 6 and figure 1**
Quantitative variables	11	Explain how quantitative variables were handled in the analyses. If applicable, describe which groupings were chosen and why **Pages 7-8**
Statistical methods	12	(*a*) Describe all statistical methods, including those used to control for confounding

For peer review only

	**Pages 8-9**
	(*b*) Describe any methods used to examine subgroups and interactions<br>**Pages 8-9**
	(*c*) Explain how missing data were addressed<br>**Figure 1, pages 8**
	(*d*) *Cohort study*—If applicable, explain how loss to follow-up was addressed<br>**Page 8**<br>*Case-control study*—If applicable, explain how matching of cases and controls was addressed<br>*Cross-sectional study*—If applicable, describe analytical methods taking account of sampling strategy
	(*e*) Describe any sensitivity analyses

Continued on next page

For peer review only

For peer review only - http://bmjopen.bmj.com/site/about/guidelines.xhtml

**Results**		
Participants	13*	Report numbers of individuals at each stage of study—eg numbers potentially eligible, examined for eligibility, confirmed eligible, included in the study, completing follow-up, and analysed<br>**Not applicable**
		(b) Give reasons for non-participation at each stage<br>**Not applicable**
		(c) Consider use of a flow diagram<br>**Figure 1**
Descriptive data	14*	Give characteristics of study participants (eg demographic, clinical, social) and information on exposures and potential confounders<br>**Table 1, page 8, lines 218-225**
		(b) Indicate number of participants with missing data for each variable of interest<br>**Figure 1 and table 1**
		(c) *Cohort study*—Summarise follow-up time (eg, average and total amount)<br>**Page 8, lines 218-225, table 1**
Outcome data	15*	*Cohort study*—Report numbers of outcome events or summary measures over time<br>**page 8, lines 218-220, table 1**
		Case-control study—Report numbers in each exposure category, or summary measures of exposure
		Cross-sectional study—Report numbers of outcome events or summary measures
Main results	16	Give unadjusted estimates and, if applicable, confounder-adjusted estimates and their precision (eg, 95% confidence interval). Make clear which confounders were adjusted for and why they were included<br>**Figures 2 and 3**
		(*b*) Report category boundaries when continuous variables were categorized<br>**Table 1**
		(*c*) If relevant, consider translating estimates of relative risk into absolute risk for a meaningful time period
Other analyses	17	Report other analyses done—eg analyses of subgroups and interactions, and sensitivity analyses<br>**Page 7, lines 206-211 and page 11, lines 303-306**
**Discussion**		
Key results	18	Summarise key results with reference to study objectives<br>**Page 9-10, lines 254-265**
Limitations	19	Discuss limitations of the study, taking into account sources of potential bias or imprecision. Discuss both direction and magnitude of any potential bias<br>**Pages 11-12 lines 319-332**
Interpretation	20	Give a cautious overall interpretation of results considering objectives, limitations, multiplicity of analyses, results from similar studies, and other relevant evidence<br>**Pages 10-11, lines 267-308**
Generalisability	21	Discuss the generalisability (external validity) of the study results
**Other information**		
Funding	22	Give the source of funding and the role of the funders for the present study and, if applicable, for the original study on which the present article is based<br>**Yes**

For peer review only

*Give information separately for cases and controls in case-control studies and, if applicable, for exposed and unexposed groups in cohort and cross-sectional studies.

Note: An Explanation and Elaboration article discusses each checklist item and gives methodological background and published examples of transparent reporting. The STROBE checklist is best used in conjunction with this article (freely available on the Web sites of PLoS Medicine at http://www.plosmedicine.org/, Annals of Internal Medicine at http://www.annals.org/, and Epidemiology at http://www.epidem.com/). Information on the STROBE Initiative is available at www.strobe-statement.org.

For peer review only

For peer review only - http://bmjopen.bmj.com/site/about/guidelines.xhtml

BMJ Open

Intermarriage and COVID-19 mortality among immigrants. A population-based cohort study from Sweden

Journal:	*BMJ Open*
Manuscript ID	bmjopen-2021-048952.R2
Article Type:	Original research
Date Submitted by the Author:	15-Jun-2021
Complete List of Authors:	Aradhya, Siddartha ; Stockholm University, Stockholm University Demography Unit<br>Branden, Maria; Linköping University, The Institute for Analytical Sociology (IAS); Stockholm University, Stockholm University Demography Unit<br>Drefahl, Sven; Stockholm University, Sociology<br>Obucina, Ognjen; Institut national d'études démographiques<br>Andersson, Gunnar ; Stockholm University, Department of Sociology<br>Rostila, Mikael ; Stockholm University, Department of Public Health Sciences<br>Mussino, Eleonora; Stockholm University, Stockholm University Demography Unit<br>Juárez , Sol Pia; Stockholm University, Department of Public Health Sciences
**Primary Subject Heading**:	Public health
Secondary Subject Heading:	Global health, Public health, Sociology, Infectious diseases
Keywords:	COVID-19, PUBLIC HEALTH, Demography < TROPICAL MEDICINE, EPIDEMIOLOGY

SCHOLARONE™
Manuscripts

I, the Submitting Author has the right to grant and does grant on behalf of all authors of the Work (as defined in the below author licence), an exclusive licence and/or a non-exclusive licence for contributions from authors who are: i) UK Crown employees; ii) where BMJ has agreed a CC-BY licence shall apply, and/or iii) in accordance with the terms applicable for US Federal Government officers or employees acting as part of their official duties; on a worldwide, perpetual, irrevocable, royalty-free basis to BMJ Publishing Group Ltd ("BMJ") its licensees and where the relevant Journal is co-owned by BMJ to the co-owners of the Journal, to publish the Work in this journal and any other BMJ products and to exploit all rights, as set out in our licence.

The Submitting Author accepts and understands that any supply made under these terms is made by BMJ to the Submitting Author unless you are acting as an employee on behalf of your employer or a postgraduate student of an affiliated institution which is paying any applicable article publishing charge ("APC") for Open Access articles. Where the Submitting Author wishes to make the Work available on an Open Access basis (and intends to pay the relevant APC), the terms of reuse of such Open Access shall be governed by a Creative Commons licence – details of these licences and which Creative Commons licence will apply to this Work are set out in our licence referred to above.

Other than as permitted in any relevant BMJ Author's Self Archiving Policies, I confirm this Work has not been accepted for publication elsewhere, is not being considered for publication elsewhere and does not duplicate material already published. I confirm all authors consent to publication of this Work and authorise the granting of this licence.

For peer review only

Intermarriage and COVID-19 mortality among immigrants. A population-based cohort study from Sweden

Siddartha Aradhya[1], Maria Brandén[1,2], Sven Drefahl[1], Ognjen Obućina[3], Gunnar Andersson[1], Mikael Rostila[4,5], Eleonora Mussino[1], Sol P. Juárez[4,5*]

1. Stockholm University Demography Unit, Stockholm University, 106 91 Stockholm, Sweden

2. Institute for Analytical Sociology, Linköping University, 601 74 Norrköping, Sweden

3. Institut national d'études démographiques (INED), 9 cours des Humanités, 93322 Aubervilliers, France

4. Department of Public Health Sciences, Stockholm University, 106 91 Stockholm, Sweden

5. Centre for Health Equity Studies (CHESS), Stockholm University/Karolinska Institute, Stockholm, Sweden

[*] Corresponding author: Sol P. Juárez, sol.juarez@su.se Department of Public Health Sciences, Stockholm University, SE-106 91, Stockholm, Sweden

Keywords: COVID-19; immigrants; language barriers; mortality; Sweden

Word count. 3324

For peer review only - http://bmjopen.bmj.com/site/about/guidelines.xhtml

Intermarriage and COVID-19 mortality among immigrants. A population-based cohort study from Sweden

Abstract

Objectives. To evaluate the role of language proficiency and understanding of the healthcare system and health recommendations in explaining excess COVID-19 mortality among immigrants.

Design. Cohort study with follow-up between March 12, 2020 and February 23, 2021.

Setting. Swedish register-based study on all residents in Sweden.

Participants. 3,963,356Swedish residents in co-residential unions who were 30 years of age or older and alive on March 12, 2020 and living in Sweden in December 2019.

Outcome measures. Cox regression models were conducted to assess the association between different constellations of immigrant-native couples (proxy for language proficiency and institutional awareness) and COVID-19 mortality and all other causes of deaths (2019 and 2020). Models were adjusted for relevant confounders.

Results. Compared to Swedish-Swedish couples (1.18 deaths per thousand person-years), both immigrants partnered with another immigrant and a native showed excess mortality for COVID-19 (HR 1.43; 95%CI 1.29, 1.58 and HR 1.24; 95%CI 1.10, 1.40, respectively) which translates to 1.37 and 1.28 deaths per thousand person-years. Moreover, similar results are found for natives partnered with an immigrant (HR 1.15; 95%CI 1.02, 1.29) which translates to 1.29 deaths per thousand person-years. Further analysis shows that immigrants from both high and low-middle income countries experience excess mortality also when partnered with a Swede. However, having a Swedish-born partner is only partially protective against COVID-19 mortality among immigrants from LMIC origins.

Conclusions. Language barriers and/or poor institutional awareness are not major drivers for the excess mortality from COVID-19 among immigrants. Rather, our study provides suggestive evidence that excess mortality among immigrants is explained by differential exposure to the virus.

Article Summary

For peer review only - http://bmjopen.bmj.com/site/about/guidelines.xhtml

- This study uses total population data with all deaths (from COVID-19 and other causes) in Sweden from March 12th 2020 to 23rd February 2021.
- We identified the origin of co-resident couples to evaluate the role of language proficiency and institutional awareness (e.g., healthcare system) in explaining excess COVID-19 mortality among immigrants.
- We compare COVID-19 mortality to all other causes of death during the pandemic and all-cause mortality one year prior to evaluate the relative impact of the pandemic within each group.
- The analyses do not include information on occupation, however the most vulnerable individuals are beyond retirement age.

For peer review only

For peer review only - http://bmjopen.bmj.com/site/about/guidelines.xhtml

International evidence has shown that immigrants and ethnic minorities are disproportionately at risk of severe COVID-19 complications and death[1–13]. In the context of an ongoing pandemic, an effort to understand the causes for why some groups are more affected is a public health priority.[14–17] Among these groups, excess mortality has been suggested to be the result of differential exposure (e.g., high-risk occupations or overcrowded accommodation), susceptibility (e.g., pre-existing conditions), and language barriers and access to healthcare.[18–20] Recent studies, however, suggest that immigrants and minorities maintain an excess mortality even after controlling for socio-economic status and housing conditions.[3,21] Yet there is paucity of evidence on the role of language barriers and institutional awareness in explaining the COVID-19 excess mortality experienced by immigrants.

Sweden took a distinct approach to dealing with the COVID-19 pandemic as compared to other Western countries by not implementing lock downs or mask mandates and instead relied largely on recommendations. The authorities justified implementing a relatively less rigid approach by arguing that Swedes have a high level of trust in their institutions and as such follow governmental recommendations.[22,23] The strategy relied primarily on public health advice (regarding hygiene routines, social distancing and suspension from work, school or daycare in case of minor symptoms) *in lieu* of mandates which are not permitted under Swedish law.[22] The effectiveness of the adopted strategy strongly depends on the ability of all members of the society to understand the recommendations, which is a basic condition for their adherence. Under this rationale, it is unsurprising that excess mortality observed among immigrants[24]—especially concentrated among those with more distant origins—could be interpreted as a consequence of lower adherence to recommendations and/or related factors.

More specifically, it has been argued that immigrants may, as a result of inadequate language proficiency and institutional awareness, have a poor understanding of the healthcare system and of the recommendations during the COVID-19 pandemic putting them at higher risk of exposure and vulnerability to the virus. Analyzing intermarried immigrants present a unique opportunity to generate evidence for this explanation.

For peer review only - http://bmjopen.bmj.com/site/about/guidelines.xhtml

Within the field of immigrant integration, intermarriage has been considered the ultimate stage of acculturation[25–27] and is, both, a marker of and facilitator for integration. Marrying a native is strongly related to language abilities, knowledge of the host country's institutions and social practices, as well as the ethnic composition of one's social circle. As a result, intermarriage reflects the narrowing of socio-cultural distance between ethnic Swedes and immigrants, which renders it an ideal measure to evaluate the role of understanding and awareness of recommendations from Swedish authorities as explanations for the excess COVID-19 mortality among immigrants.

If understanding and awareness of recommendations (language *in primis*) explain the excess mortality, immigrants partnered with a Swede and, in particular, Swedes partnered with an immigrant should have similar mortality to Swedes partnered with a Swede. To this end, the aim of this study is to examine the association between socio-cultural integration and COVID-19 mortality by examining native-immigrant couple dyads—a well-regarded measure that has been shown to be a marker and facilitator of integration.

Methods

Study population

An observational cohort study was conducted using Swedish register data. The study includes all Swedish residents who were 30 years of age or older and were cohabiting with another adult who was at least 30 years of age and alive on March 12th, 2020, residing in Sweden in December 2019 (n= 4,019,418). This age restriction was established to ensure co-resident individuals were family members and not flat mates. The follow-up period was March 12, 2020 up until February 23, 2021. We excluded individuals who had not lived in Sweden in the two prior years (n=40,515), because they could not be linked to all records of data. In addition, we excluded individuals with missing data on country of birth (n=142) and income (n=15,405) of either partner. The final study population consists of 3,963,356 individuals (18.5% immigrants) (figure 1).

Patient and public involvement

For peer review only - http://bmjopen.bmj.com/site/about/guidelines.xhtml

No patient involved

Data

We use information from several Swedish administrative registers linked through personal identity numbers that are unique to each person with legal residence in Sweden. Data on deaths were retrieved from the Cause of Death Register. Socioeconomic and demographic variables (income, education, number of children, and region of residence) were drawn from the Longitudinal integrated database for health insurance and labor market studies (LISA), and residential information (type and crowdedness of the dwelling) were drawn from the Dwelling register. All covariates in our study are time-constant and either measured at the end of 2019 (all variables at the household and neighborhood level) or 2018 (highest education attained, sum of the individual net incomes of the two co-resident adults, total number of individuals in the household under 30). Information on age, sex, country of birth and immigrant density in the neighborhood stem from the Total Population Register. It is important to note that all individuals registered in Sweden are entitled to health care access.

Study variables

COVID-19 mortality was identified by the Swedish National Board of Health and Welfare (*Socialstyrelsen*), the agency responsible for the cause of death register. COVID-19 mortality was identified using the following ICD codes for the underlying cause of death: U07.1 (3,915 deaths), U07.2 (127 deaths), and B34.2 (2 deaths); for 522 more deaths ICD codes U07.1, U07.2 or B34.2 were listed as contributing causes of death, excluding mortality from all other causes of death (30,374 deaths). Given the timeliness of the data, the assignment of the underlying cause of death should be understood as preliminary.

Immigrant-native couple types were created by combining information from the dwelling and the total population register to create the couple type which include two individuals of at least 30 years of age co-residing in the same household. The variable is classified into the following four ego-partner categories using information on country of birth: (i) native-native, (ii) native-immigrant, (iii) immigrant-native, and (iv) immigrant-immigrant.

For peer review only - http://bmjopen.bmj.com/site/about/guidelines.xhtml

We chose (i) native-native couples as the reference group for our analyses as they a) constitute the largest category among all groups considered and b) represent the institutional awareness and language of the host population from which we expect other groups to deviate. We further disaggregated the groups by immigrant's origins defined according to the World Bank classification based on the Gross National Incomes (GNI) per capita using the WB Atlas method[28] as low-middle and high income countries.

We derived individual income and calculated the sum of the two partners' net incomes (household), categorized into tertiles based on all adult residents of Sweden. We derived education data from Swedish educational registers and categorize our population into four categories; those with primary schooling, secondary schooling, post-secondary education, and those with missing information on education. Missing information on education is generally very low but 88% of those with missing education are immigrants. We additionally performed multiple imputation to test how the missing values for education impact our results (see Appendix figure S1). In addition, we dropped all missing categories to assess whether the distributions of covariates were affected by missing values (see Appendix table S1). From the Swedish Dwelling register we accessed information on size of the dwelling and a unique dwelling code which enables us to link individuals who live together in a household and determine co-residence. In addition to define couples, we use this information to create: the number of individuals per square meter in the household (with a separate category for a small group of individuals, due to missing information on square meters in some detached houses), the number of individuals living in the household under the age of 30, and dwelling type (multi-family, single family, or care home). We include in our model also the share of immigrants in the local neighborhood, DeSO (a smaller subdivision of administrative areas based on demographic characteristics produced by the Swedish administrative statistics).

Statistical analysis

We conducted Cox proportional hazards regressions (using age as the timescale) to estimate Hazard Ratios (HR) and 95% Confidence Intervals (CI) for the association between immigrant-native couple type and COVID-19 mortality. Individuals exited the

For peer review only - http://bmjopen.bmj.com/site/about/guidelines.xhtml

study by (1) dying between March 12, 2020 and February 23, 2021, or (2) being alive on February 23, 2021. We estimated two separate regressions estimating the cause-specific hazard of dying from COVID-19, right-censoring all individuals that die from other causes and (2) the cause-specific hazard of dying from other causes than COVID-19, right-censoring all individuals that die from COVID-19. In addition, we conducted Cox regressions for dying from all causes of death that occurred between March 12, 2019 and February 23, 2020 (31,653 deaths)—the same period we observe COVID-19 deaths twelve contiguous months prior to the start of the pandemic. Since mortality from COVID-19 and other causes of deaths during the study window are not fully independent of each other, our estimates for all-cause mortality between March 12, 2019 and February 23, 2020 were used to evaluate the robustness of our estimates for mortality during the pandemic. In addition, the comparison between all-cause mortality one year prior and mortality from causes other than COVID-19 during the pandemic, by immigrant-native couple type, will allow us to examine whether the latter has a distinctive role in relation to the pandemic.

Two models were estimated: 1) a simple model with age as the time scale adjusted for sex and 2) the same model with further adjustments. In the latter analysis we adjust for education, neighborhood immigrant density, and region of residence fixed-effects that are confounders, as well as factors that are on the causal pathway that have been previously used to explain the excess mortality of immigrants (i.e, number of individuals in the household below the age of 30, dwelling type, square meters per person in the dwelling, household income). In addition, we conducted two sensitivity analyses. One in which we examined the partner's origin among Swedish-migrant partnerships (high or low-middle income country) to check whether patterns are consistent across groups and, a second, in which we excluded all individuals born in Sweden with at least one foreign-born parent. All analyses were conducted using Stata Statistical Software: Release 16 (StataCorp LP, College Station, Texas).

This study was approved by the Central Ethical Review Board in 2020 (Dnr 2020-02199).

Results

For peer review only - http://bmjopen.bmj.com/site/about/guidelines.xhtml

During the 3,759,610 person-years of observation, 4,564 COVID-19 deaths occurred in our study population between March 12, 2020 and February 23, 2021. Table 1 shows the distribution of population at risk and deaths by all covariates. In our population, 6.09% of individuals are in immigrant-native couples. Native-native couples show the lowest deaths per thousand person-years (1.18/1000), whereas all other couple types show higher death rates of COVID-19 mortality (approximately 1.3-1.4 per thousand person-years). Of the study population, 6.55% of COVID-19 deaths were attributed to native-immigrant mixed couples and 14.04% to immigrant-immigrant couples.

Figure 2 displays mortality risks from COVID-19, all other causes of death during the pandemic, and all-causes of death one year prior across couple types with native-native as the reference (see Appendix table S2 for estimates). Panel A presents models adjusted for age and sex and panel B presents the estimates including adjustments. In A, individuals in immigrant-immigrant couples show the highest HR of dying from COVID-19 (HR 2.47; 95%CI: 2.27, 2.69) and those in native-native couples the lowest (reference group) while natives (HR 1.40; 95%CI: 1.25, 1.58) and immigrants (HR 1.50; 95%CI: 1.33, 1.69) in mixed couples showed intermediate mortality levels. All-causes of death in the year prior to the pandemic and all-other causes of death during the pandemic show little differences between couple constellations. After adjustments (panel B), differences across groups attenuate, but the gradient in COVID-19 mortality remains. Individuals in immigrant-immigrant couples still display the highest HR of dying from COVID-19 relative to the reference group (HR 1.43; 95%CI: 1.29, 1.58), followed by those in immigrant-native couples (HR 1.24; 95%CI: 1.10, 1.40) and native-immigrant couples (HR 1.15; 95%CI: 1.02, 1.29). It is important to note that there is no statistically significant difference in the risk of COVID-19 mortality between immigrant-native and immigrant-immigrant couples. An opposite gradient is observed with respect to all-cause mortality in the year prior to the pandemic and all other causes of death during the pandemic, with immigrant-immigrant couples displaying the lowest mortality.

Figure 3 is an extension of figure 2 disaggregating the immigrant population by income level of their country of birth (see Appendix table S3 for estimates). In panel A, there are

For peer review only - http://bmjopen.bmj.com/site/about/guidelines.xhtml

elevated HRs across all origin groups relative to native-native couples. Immigrants in LMIC-immigrant couples display the highest HR relative to the reference group (HR 3.60; 95%CI 3.25, 3.99) followed by those in LMIC-native couples (HR 1.91; 95%CI 1.42, 2.57). Natives in native-immigrant couples, where HIC & LMIC have been pooled, (HR 1.41; 95%CI 1.25, 1.58), HIC-native (HR 1.44; 95%CI 1.27, 1.64), and immigrants in HIC-immigrant (HR 1.54; 95%CI 1.34, 1.77) couples display relatively similar HRs. After adjustment (panel B), all groups with an immigrant still display higher HRs relative to native-native couples, but at a lower level. LMIC-immigrant couples experience a particularly strong reduction in their HRs (HR 1.84; 95%CI 1.62, 2.09). Compared to LMIC-native couples, their HR remains slightly higher (HR 1.35; 95%CI 1.00, 1.82). We also find that HIC-native (HR 1.23; 95%CI 1.08, 1.40) and HIC-immigrant (HR 1.11; 95%CI 0.97, 1.28) couples display higher HRs relative to the reference group.

Sensitivity analyses showed that disaggregating the origin of immigrants in native-immigrant partnerships (appendix figure S2) and excluding second-generation Swedes from the Swedish-born population (appendix figure S3) produce no further differentials in mortality risks.

Discussion

Our study shows that immigrants have excess COVID-19 mortality regardless of the origin of their partner, where having a Swedish-born partner is only partially protective against COVID-19 mortality among immigrants from low-middle income countries. These findings challenge hypotheses that poor language proficiency and institutional awareness are major contributing factors explaining the excess mortality from COVID-19 among immigrants.

Language has been considered a vital component of integration and relevant for accessing information for other types of medical treatments and health outcomes[29]. Given that the COVID-19 pandemic is a unique occurrence that was accompanied by a global diffusion of information, one can argue that even those with presumably little Swedish proficiency have been exposed to recommendations offered in their native languages

For peer review only - http://bmjopen.bmj.com/site/about/guidelines.xhtml

from either public health officials from their countries of origin or via other international channels. In fact, the information that they may have received from international sources may be more relevant for specific immigrant populations, for example, how to best protect oneself when observing cultural or religious practice. At the same time the Swedish authorities have translated information to other languages, albeit not cultural adapted. However, in the first stage of the pandemic, the authorities were delayed in releasing information in all languages and, at least in Stockholm, we found no group differences among intermarried groups and immigrant-immigrant couples (see appendix figure S4). This highlights how language is no straightforward factor in mitigating the burden of COVID-19 on immigrant populations.

Prior studies in clinical settings have shown that access to a medical interpreter is associated with better health access and outcomes.[29] Although we cannot test this aspect directly, our study shows that immigrants partnered with Swedes are slightly protected with respect to COVID-19 mortality. This suggests that lower language barriers may indeed be relevant with respect to interacting with the healthcare system. To the best of our knowledge, however, we are unaware of any additional provisions provided to non-Swedish speakers at hospitals or clinics during the pandemic.

Beyond allowing us to disentangle the role of language barriers and lack of understanding of the healthcare system and recommendations in explaining the excess COVID-19 mortality among immigrants, our study provides suggestive evidence that differential exposure to the virus and not susceptibility is the culprit.[20] Although it is true that Swedes partnered with a Swede show the lowest mortality, those partnered with an immigrant experience higher COVID-19 mortality. Given that Swedes do not experience language barriers or lack institutional awareness and that biological susceptibility cannot be transmitted between partners, Swedes partnered with immigrants are likely to be at either higher exposure to the virus or impacted by the social susceptibility of their partner. Swedes in mixed partnerships may be exposed to similar social environments and/or risk factors as immigrants that place them at a higher risk of exposure as compared to Swedes partnered with another Swede. For example, they may have more transnational contacts or are impacted by some of the same social risk factors as immigrants. Moreover, the

For peer review only - http://bmjopen.bmj.com/site/about/guidelines.xhtml

higher mortality experienced by natives partnered with immigrants could be related to the disadvantages faced by the immigrant partner either via discrimination or a higher exposure to the pandemic (e.g., having a frontline or precarious occupation). Albeit this type of exposure in mixed partnerships might not be at the same level as for immigrants partnered with another immigrant (as our results with a gradient in mortality suggests).

In this study, immigrants in different family constellations show higher levels of COVID-19 deaths than the majority of natives, after adjustment for a wide range of individual- and contextual-level factors, including education, income, housing conditions, and neighborhood immigrant density. This set of adjustments also partly accounts for a number of socially patterned chronic health conditions and comorbidities, e.g., insulin resistance, hypertension, smoking and obesity, which have been suggested as risk factors for severe cases of COVID-19.[17]

This study has a number of contributions and strengths, First, this is the only study to date to examine socio-cultural integration as the mechanism behind the disproportionate burden that COVID-19 has placed on immigrant communities, by comparing the mortality of native-native, immigrant-native, and immigrant-immigrant couples in Sweden. A major strength of our study is that we have complete coverage of the total population and all deaths in Sweden from the start of the pandemic until February 2021 for both COVID-19 and remaining causes of death. Thus, our analysis does not suffer from selection into our study population. We have similar high-quality data for the year prior to the pandemic, which allows for an unbiased comparison of mortality patterns between the two years. The comparison between couple types with respect to mortality from all other causes during the pandemic and all-cause mortality in the year prior strengthens our findings as it demonstrates that the excess mortality from COVID-19 is observed in couples that show no excess mortality from other causes before and during the pandemic.

Despite its strengths, this study has also some limitations that are worth mentioning. Although the Swedish population registers hold high quality and have many advantages, they capture de jure rather than de facto characteristics of individuals. With respect to our

For peer review only - http://bmjopen.bmj.com/site/about/guidelines.xhtml

measure of partnership, 82% of the couples in our data are either married or have shared children while the remaining 18% co-reside unmarried without common children. However, non-marital cohabitation is very common in Sweden, while flat-sharing is not[30], and particularly among individuals above their 30s. It is therefore fair to assume that a substantive share in this remaining group is indeed cohabiting in an amorous relationship, a group that is often overlooked in international studies of health and mortality because of the lack of data. A final limitation is that we do not include information on occupation. Specifically, it has been hypothesized that immigrants are more likely to work in 'front-line' occupations; however, recent research in Sweden has shown that occupational exposure is not a risk factor for COVID-19 mortality.[31] Furthermore, it is worth noting that a majority of deaths are occurring among retired individuals with no attachment to the labor market.

In conclusion, our study shows that being partnered with a native does not close the gap in COVID-19 mortality with natives even after adjusting for a wide range of possible confounders on both individual- couple- and residential level. As such, these findings show that lack of awareness of the Swedish recommendations and language barriers are not major drivers for the excess COVID-19 mortality of immigrants. At the same time, the fact that Swedes partnered with immigrants also show excess mortality compared to Swedish couples, suggests that excess mortality among immigrants is explained by differential exposure to the virus.

For peer review only - http://bmjopen.bmj.com/site/about/guidelines.xhtml

Ethical approval: The analyses have been approved by the Swedish ethical-vetting authority, Dnr 2020-02199.

Contributorship statement: SA, MB and SJ conceived the study and were responsible for the planning. GA and MB provided the data, MB analyzed the data, SA, SJ, SD, EM, MB designed the analysis with contributions from OO, MR, GA. SA, SJ, SD, EM, MB, OO, MR, GA contributed to the interpretation of the data. SA and SJ drafted the manuscript with substantive contributions from EM, SD, MB, OO, MR, GA. All authors approved the final version of the manuscript.

Data sharing: This study is produced under the Swedish Statistics Act, where privacy concerns restrict the availability of register data for research. Aggregated data can be made available by the authors, conditional on ethical vetting. The authors access the individual-level data through Statistics Sweden's micro-online access system MONA.

Competing Interest statement: No, there are no competing interests for any author

Acknowledgements: We thank Thomas Niedomysl of Region Halland, Petra Westin of the National Board of Health and Welfare, and Simon Kurt of Statistics Sweden for their invaluable role in providing the data.

Funding: The Swedish Research Council for Health, Working Life and Welfare (FORTE), grant numbers 2016-07115, 2016-07105, 2016-07128, 2019-00603, The Swedish Research Council (VR), grant number 2018-01825 and The Swedish Foundation for Humanities and Social Sciences (Riksbankens Jubileumsfond), grant M18-0214:1.

For peer review only

References:

1. Pan, D. *et al.* The impact of ethnicity on clinical outcomes in COVID-19: A systematic review. *EClinicalMedicine* **23**, 100404 (2020).
2. Hayward, S. E. *et al.* Clinical outcomes and risk factors for COVID-19 among migrant populations in high-income countries: A systematic review. *Journal of Migration and Health* **3**, 100041 (2021).
3. Drefahl, S. *et al.* A population-based cohort study of socio-demographic risk factors for COVID-19 deaths in Sweden. *Nat Commun* **11**, 5097 (2020).
4. Aldridge, R. W. *et al.* Black, Asian and Minority Ethnic groups in England are at increased risk of death from COVID-19: indirect standardisation of NHS mortality data. *Wellcome Open Res* **5**, 88 (2020).
5. Rostila, M. *et al.* Disparities in COVID-19 deaths by country of birth in Stockholm, Sweden: A total population based cohort study. *American Journal of Epidemiology* (2021) doi:10.1093/aje/kwab057.
6. Niedzwiedz, C. L. *et al.* Ethnic and socioeconomic differences in SARS-CoV-2 infection: prospective cohort study using UK Biobank. *BMC Med* **18**, 160 (2020).
7. Ross, J., Diaz, C. M. & Starrels, J. L. The Disproportionate Burden of COVID-19 for Immigrants in the Bronx, New York. *JAMA Intern Med* (2020) doi:10.1001/jamainternmed.2020.2131.
8. Millett, G. A. *et al.* Assessing Differential Impacts of COVID-19 on Black Communities. *Annals of Epidemiology* S1047279720301769 (2020) doi:10.1016/j.annepidem.2020.05.003.
9. Williamson, E. J. *et al.* Factors associated with COVID-19-related death using OpenSAFELY. *Nature* **584**, 430–436 (2020).

For peer review only

For peer review only - http://bmjopen.bmj.com/site/about/guidelines.xhtml

10. Indseth, T. *et al.* COVID-19 among immigrants in Norway, notified infections, related hospitalizations and associated mortality: A register-based study. *Scand J Public Health* **49**, 48–56 (2021).

11. Raharja, A., Tamara, A. & Kok, L. T. Association Between Ethnicity and Severe COVID-19 Disease: a Systematic Review and Meta-analysis. *J. Racial and Ethnic Health Disparities* (2020) doi:10.1007/s40615-020-00921-5.

12. Staetsky, L. D. Elevated Jewish Mortality from Coronavirus in England and Wales: An Epidemiological and Demographic Detective Story. *Cont Jewry* (2021) doi:10.1007/s12397-021-09366-2.

13. Vanthomme, K., Gadeyne, S., Lusyne, P. & Vandenheede, H. A population-based study on mortality among Belgian immigrants during the first COVID-19 wave in Belgium. Can demographic and socioeconomic indicators explain differential mortality? *SSM - Population Health* **14**, 100797 (2021).

14. Statement by the EUPHA Migrant and ethnic minority health section on COVID-19 – CALL FOR ACTION: Reducing the impact of the coronavirus crisis on those 'left behind' – Disadvantaged Migrants and Ethnic Minorities (MEM). (2020).

15. Pareek, M. *et al.* Ethnicity and COVID-19: an urgent public health research priority. *The Lancet* **395**, 1421–1422 (2020).

16. Bhala, N., Curry, G., Martineau, A. R., Agyemang, C. & Bhopal, R. Sharpening the global focus on ethnicity and race in the time of COVID-19. *The Lancet* **0**, (2020).

17. Khunti, K., Singh, A. K., Pareek, M. & Hanif, W. Is ethnicity linked to incidence or outcomes of covid-19? *BMJ* **369**, (2020).

For peer review only - http://bmjopen.bmj.com/site/about/guidelines.xhtml

18. Diaz, E. *et al. Situational brief: Migration and Covid-19 in Scandinavian countries*. https://www.migrationandhealth.org/migration-covid19-briefs (2020).

19. Diderichsen, F. How did Sweden Fail the Pandemic? *Int J Health Serv* 002073142199484 (2021) doi:10.1177/0020731421994848.

20. Katikireddi, S. V. *et al.* Unequal impact of the COVID-19 crisis on minority ethnic groups: a framework for understanding and addressing inequalities. *J Epidemiol Community Health* jech-2020-216061 (2021) doi:10.1136/jech-2020-216061.

21. Rostila, M. *et al.* Disparities in covid-19 deaths by country of birth in Stockholm, Sweden: A total population based cohort study. *Stockholm Research Reports in Demography. Preprint.* (2020) doi:https://doi.org/10.17045/sthlmuni.12852854.v1.

22. Pierre, J. Nudges against pandemics: Sweden's COVID-19 containment strategy in perspective. *Policy and Society* **39**, 478–493 (2020).

23. Paterlini, M. 'Closing borders is ridiculous': the epidemiologist behind Sweden's controversial coronavirus strategy. *Nature* **580**, 574–574 (2020).

24. Folkhälsomydigheten. *Veckorapport om covid-19, vecka 15*. https://www.folkhalsomyndigheten.se/globalassets/statistik-uppfoljning/smittsamma-sjukdomar/veckorapporter-covid-19/2020/covid-19-veckorapport-vecka-15.pdf (2020).

25. Alba, R. D. & Golden, R. M. Patterns of Ethnic Marriage in the United States. *Social Forces* **65**, 202–223 (1986).

26. Lieberson, S. & Waters, M. C. Ethnic groups in flux: the changing ethnic responses of American whites. *The Annals of the American Academy of Political and Social Science* **487**, 79–91 (1986).

For peer review only - http://bmjopen.bmj.com/site/about/guidelines.xhtml

27. Kalmijn, M. Intermarriage and Homogamy: Causes, Patterns, Trends. *Annual Review of Sociology* **24**, 395–421 (1998).

28. The World Bank. GNI, Atlas method (current US$) | Data. *DataBank* https://data.worldbank.org/indicator/NY.GNP.ATLS.CD.

29. Flores, G. The Impact of Medical Interpreter Services on the Quality of Health Care: A Systematic Review. *Med Care Res Rev* **62**, 255–299 (2005).

30. SCB. *Ensam, med partner eller kompisboende Vad säger hushållsregistret?* https://www.scb.se/contentassets/cfe7690018d741798939bd8a6d087219/be0701_2015i2018_br_be51br1901.pdf (2019).

31. Billingsley, S. *et al.* Deaths in the frontline: Occupation-specific COVID-19 mortality risks in Sweden. *Stockholm Research Reports in Demography. Preprint.* (2020) doi:DOI: 10.17045/sthlmuni.12816065.v2.

For peer review only

For peer review only - http://bmjopen.bmj.com/site/about/guidelines.xhtml

Table 1. Description of the study population, number, proportion of deaths and death rates

	Total		COVID-19		Other cause of death		Exposure time in years	COVID-19 deaths per 1000 years
	N at March 12, 2020	%	Deaths March 12, 2020 – February 23, 2021	%	Deaths March 12, 2020 – February 23, 2021	%		
**Couple type**								
Native-native	2,984,648	75.31	3,330	72.96	24,846	81.80	2,830,382	1.18
Native-immigrant	245,124	6.18	299	6.55	1,739	5.73	232,563	1.29
Immigrant-native	241,185	6.09	294	6.44	1,507	4.96	228,949	1.28
Immigrant-immigrant	492,399	12.42	641	14.04	2,282	7.51	467,716	1.37
**Couple type, detailed**								
Native-native	2,984,648	75.31	3,330	72.96	24,846	81.80	2,830,382	1.18
Native-immigrant	245,124	6.18	299	6.55	1,739	5.73	232,563	1.29
HIC-native	140,281	3.54	249	5.46	1,294	4.26	132,929	1.87
HIC-immigrant	121,148	3.06	215	4.71	1,170	3.85	114,765	1.87
LMIC-native	100,904	2.55	45	0.99	213	0.70	96,020	0.47
LMIC-immigrant	371,251	9.37	426	9.33	1,112	3.66	352,951	1.21
**Sex**								
Male	1,992,097	50.26	3,145	68,91	19,003	62.56	1,887,523	1.67
Woman	1,971,259	49.74	1,419	31.09	11,371	37.44	1,872,087	0.76
**Education**								
Primary	589,969	14.89	1,810	39.66	11,495	37.84	555,860	3.26
Secondary	1,687,832	42.59	1,732	37.95	12,069	39.73	1,601,553	1.08
Post-secondary	1,651,905	41.68	900	19.72	6,420	21.14	1,570,393	0.57
Missing	33,650	0.85	122	2.67	390	1.28	31,804	3.84
**Household income (tertile)**								
Lowest	1,289,265	32.53	3,544	77.65	22,358	73.61	1,216,124	2.91
Middle	1,336,367	33.72	625	13.69	5,008	16.49	1,270,568	0.49
Highest	1,337,724	33.75	395	8.65	3,008	9.90	1,272,918	0.31
**Housing type**								
Multi-family	1,283,364	32.38	2,248	49.26	11,797	38.84	1,216,054	1.85
Single-family	2,662,463	67.18	1,930	42.29	16,946	55.79	2,527,860	0.76
Care home	17,529	0.44	386	8.46	1,631	5.37	15,696	24.59
**Number of people under 30 in the household**								
0	2,079,106	52.46	4,244	92.99	28,211	92.88	1,965,558	2.16
1	614,976	15.52	180	3.94	1,191	3.92	585,276	0.31
2	870,872	21.97	85	1.86	647	2.13	829,379	0.10
3+	398,402	10.05	55	1.21	325	1.07	379,397	0.14
**m2/person in the household (crowdedness)**								
0-	325,441	8.21	325	7.12	1,463	4.82	309,194	1.05
20-	814,282	20.55	464	10.17	2,809	9.25	774,226	0.60
30-	913,084	23.04	1,240	27.17	7,232	23.81	865,941	1.43
40-	1,127,740	28.45	1,662	36.42	11,264	37.08	1,068,381	1.56

For peer review only

For peer review only - http://bmjopen.bmj.com/site/about/guidelines.xhtml

60-	750,691	18.94	861	18.87	7,468	24.59	711,339	1.21
Missing	32,118	0.81	12	0.26	138	0.45	30,530	0.39
**Share immigrants in DeSO (%)**								
0-	1,306,913	32.97	958	20.99	9,358	31.40	1,240,219	0.77
0.10-	943,691	23.81	972	21.30	7,318	24.09	895,256	1.09
0.15-	659,303	16.63	858	18.80	5,003	16.47	625,400	1.37
0.20-	556,222	14.03	765	16.76	4,575	15.06	527,391	1.45
0.30-	349,234	8.81	637	13.96	2,896	9.53	331,065	1.92
0.50-	147,993	3.73	374	8.19	1,044	3.44	140,278	2.67
**TOTAL**	3,963,356	100.0	4,564	100.0	30,374	100.0	3,759,610	1.21

Figure legends.

Figure 1. Selection flow and final sample

Figure 2. Hazard Ratios for the risk of dying from COVID-19, other causes of death during the pandemic, and all-cause mortality in the year prior to the pandemic by couple dyad. Model A is adjusted for age and sex only and model B includes full adjustment (reference group: native-native couples)

Figure 3. Hazard Ratios for the risk of dying from COVID-19, other causes of death during the pandemic, and all-cause mortality in the year prior to the pandemic by couple dyad disaggregated by LMIC and HIC immigrants. Model A is adjusted for age and sex only and model B includes full adjustment (reference group: native-native couples)

For peer review only

For peer review only - http://bmjopen.bmj.com/site/about/guidelines.xhtml

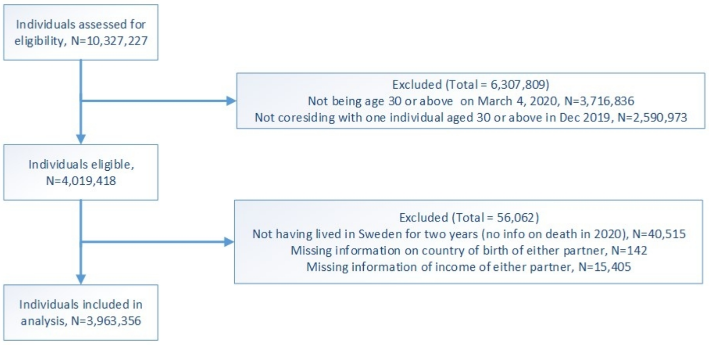

Figure 1

338x190mm (96 x 96 DPI)

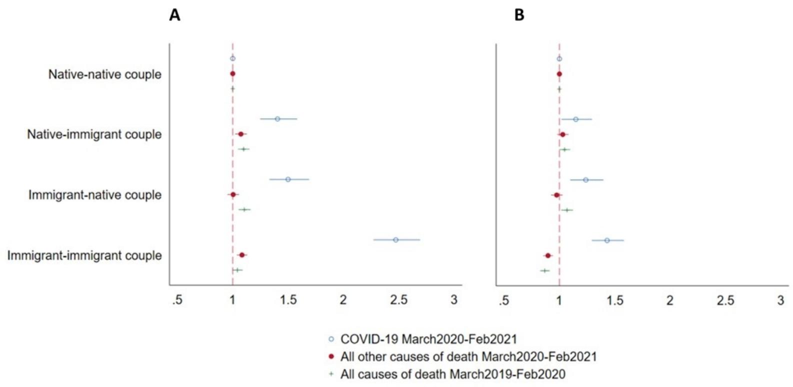

Figure 2

338x190mm (96 x 96 DPI)

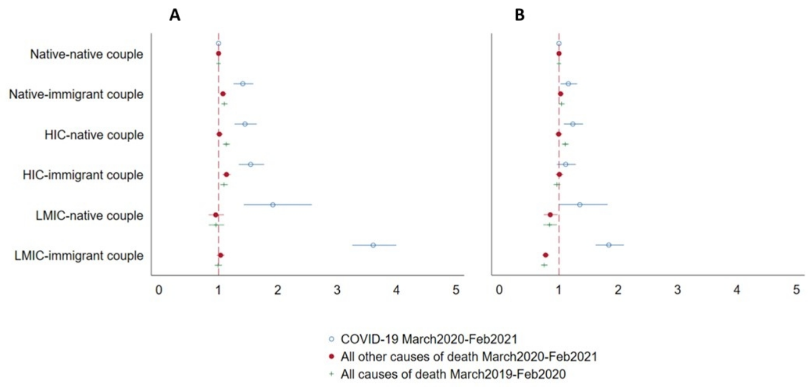

Figure 3

338x190mm (96 x 96 DPI)

Supplementary table 1: Descriptive table displaying distributions of all covariables with and without missing in education and dwelling size.

	Study population (with missing education and dwelling size)	Study population (without missing education)	Study population (without missing dwelling size)
**Couple dyads**			
Native-native	75.31	75.86	75.25
Native-immigrant	6.18	6.21	6.19
Immigrant-native	6.09	5.99	6.09
Immigrant-immigrant	12.42	11.94	12.47
**Sex**			
Man	50.26	50.21	50.26
Woman	49.74	49.79	49.74
**Education**			
Primary	14.89	15.01	14.91
Secondary	42.59	42.95	42.58
Post-secondary	41.68	42.04	41.66
Missing	0.85		0.85
**Income HH disposable Inc (tertlies)**			
1	32.53	32.22	32.58
2	33.72	33.85	33.73
3	33.75	33.93	33.69
**Housing type**			
Multi-family dwelling	32.38	32.11	32.64
Single-family dwelling	67.18	67.47	66.91
Care home	0.44	0.42	0.45
**# of individuals in the househould**			
2	52.46	52.47	52.50
3	15.52	15.50	15.53
4	21.97	22.02	21.93
5+	10.05	10.01	10.04
**Sqm per person**			
0	8.21	8.08	8.28
-20	20.55	20.50	20.71
-30	23.04	23.04	23.23
-40	28.45	28.54	28.69
-60	18.94	19.03	19.10
Missing	0.81	0.81	
**Immigrants in DeSO**			
0	32.97	33.15	32.77
.1-	23.81	23.88	23.82
.15-	16.63	16.64	16.70
.2-	14.03	14.01	14.09
.3-	8.81	8.72	8.86
.5-	3.73	3.61	3.76
**Region of residence**			
Stockholms län	21.11	21.01	21.13

For peer review only

Uppsala län	3.57	3.57	3.57
Södermanlands län	2.91	2.92	2.90
Östergötlands län	4.64	4.65	4.65
Jönköpings län	3.73	3.74	3.73
Kronobergs län	2.05	2.04	2.05
Kalmar län	2.60	2.60	2.60
Gotlands län	0.59	0.60	0.59
Blekinge län	1.68	1.68	1.67
Skåne län	13.24	13.20	13.24
Hallands län	3.59	3.60	3.59
Västra Götalands län	16.71	16.73	16.75
Värmlands län	2.89	2.88	2.89
Örebro län	2.95	2.96	2.95
Västmanlands län	2.74	2.74	2.75
Dalarnas län	2.95	2.96	2.93
Gävleborgs län	2.89	2.91	2.90
Västernorrlands län	2.56	2.57	2.56
Jämtlands län	1.29	1.30	1.28
Västerbottens län	2.75	2.76	2.73
Norrbottens län	2.56	2.57	2.54

For peer review only

Supplementary table 2: Regression results for the risk of dying from COVID-19, other causes of death during the pandemic, and all-cause mortality in the year prior to the pandemic by couple dyad

	Model 1: Figure 2A						Model 2: Figure 2B					
	COVID- 19		Other COD Mar 5, 2020- Feb 23, 2021		All COD Mar 5, 2019 – Feb 23, 2020		COVID- 19		Other COD Mar 5, 2020- Feb 23, 2021		All COD Mar 5, 2019 – Feb 23, 2020	
	HR	95% CI	HR	95% CI	HR	95% CI	HR	95% CI	HR	95% CI	HR	95% CI
**Partnership type**												
Native-native	1.00	1.00	1.00	1.00	1.00	1.00	1.00	1.00	1.00	1.00	1.00	1.00
Native-immigrant	1.40	(1.25, 1.58)	1.07	(1.02, 1.13)	1.10	(1.05, 1.15)	1.15	(1.02, 1.29)	1.03	(0.98, 1.08)	1.05	(1.00, 1.10)
Immigrant-native	1.50	(1.33, 1.69)	1.00	(0.95, 1.06)	1.11	(1.05, 1.16)	1.24	(1.10, 1.40)	0.98	(0.93, 1.03)	1.07	(1.02, 1.12)
Immigrant-immigrant	2.47	(2.27, 2.69)	1.08	(1.04, 1.13)	1.04	(1.00, 1.09)	1.43	(1.29, 1.58)	0.90	(0.85, 0.94)	0.87	(0.83, 0.91)
Years under risk	3,759,610		3,759,610		3,764,370		3,759,610		3,759,610		3,764,370	
N events	4564		30,374		31,653		4564		30,374		31,653	
N	3,963,356		3,963,356		3,966,345		3,963,356		3,963,356		3,966,345	

Model 1 includes adjustments for sex; Model 2 includes adjustments for sex, household income, education, housing type, number of individuals in the household, m^2/person in the household (crowdedness), share of immigrants in the neighborhood of residence, and region of residence

For peer review only

Supplementary table 3: Regression results for the risk of dying from COVID-19, other causes of death during the pandemic, and all-cause mortality in the year prior to the pandemic by couple dyad disaggregated by LMIC and HIC immigrants.

	Model 3: Figure 3A						Model 4: Figure 3B					
	COVID- 19		Other COD Mar 5, 2020- Feb 23, 2021		All COD Mar 5, 2019 – Feb 23, 2020		COVID- 19		Other COD Mar 5, 2020- Feb 23, 2021		All COD Mar 5, 2019 – Feb 23, 2020	
	HR	95% CI	HR	95% CI	HR	95% CI	HR	95% CI	HR	95% CI	HR	95% CI
**Partnership type**												
Native-native	1.00	1.00	1.00	1.00	1.00	1.00	1.00	1.00	1.00	1.00	1.00	1.00
Native-immigrant	1.41	(1.25, 1.58)	1.07	(1.02, 1.13)	1.10	(1.05, 1.15)	1.16	(1.03, 1.30)	1.03	(0.98, 1.08)	1.04	(0.99, 1.09)
HIC-native	1.44	(1.27, 1.64)	1.01	(0.96, 1.07)	1.13	(1.07, 1.19)	1.23	(1.08, 1.40)	0.99	(0.94, 1.05)	1.11	(1.05, 1.16)
HIC-immigrant	1.54	(1.34, 1.77)	1.13	(1.07, 1.20)	1.09	(1.03, 1.16)	1.11	(0.97, 1.28)	1.01	(0.95, 1.07)	0.97	(0.91, 1.02)
LMIC-native	1.91	(1.42, 2.57)	0.95	(0.83, 1.09)	0.96	(0.84, 1.09)	1.35	(1.00, 1.82)	0.85	(0.74, 0.98)	0.84	(0.74, 0.97)
LMIC-immigrant	3.60	(3.25, 3.99)	1.03	(0.97, 1.10)	0.99	(0.93, 1.06)	1.84	(1.62, 2.09)	0.77	(0.72, 0.83)	0.75	(0.70, 0.81)
Years under risk	3,759,610		3,759,610		3,764,370		3,759,610		3,759,610		3,764,370	
N events	4564		30,374		31,653		4564		30,374		31,653	
N	3,963,356		3,963,356		3,966,345		3,963,356		3,963,356		3,966,345	

For peer review only

Model 3 includes adjustments for sex; Model 4 includes adjustments for sex, household income, education, housing type, number of individuals in the household, m^2/person in the household (crowdedness), share of immigrants in the neighborhood of residence, and region of residence

For peer review only

Supplementary figure 1: Hazard Ratios for the risk of dying from COVID-19 by couple dyad, including estimates from sensitivity analyses using multiple imputations to adjust for missing education and only complete cases

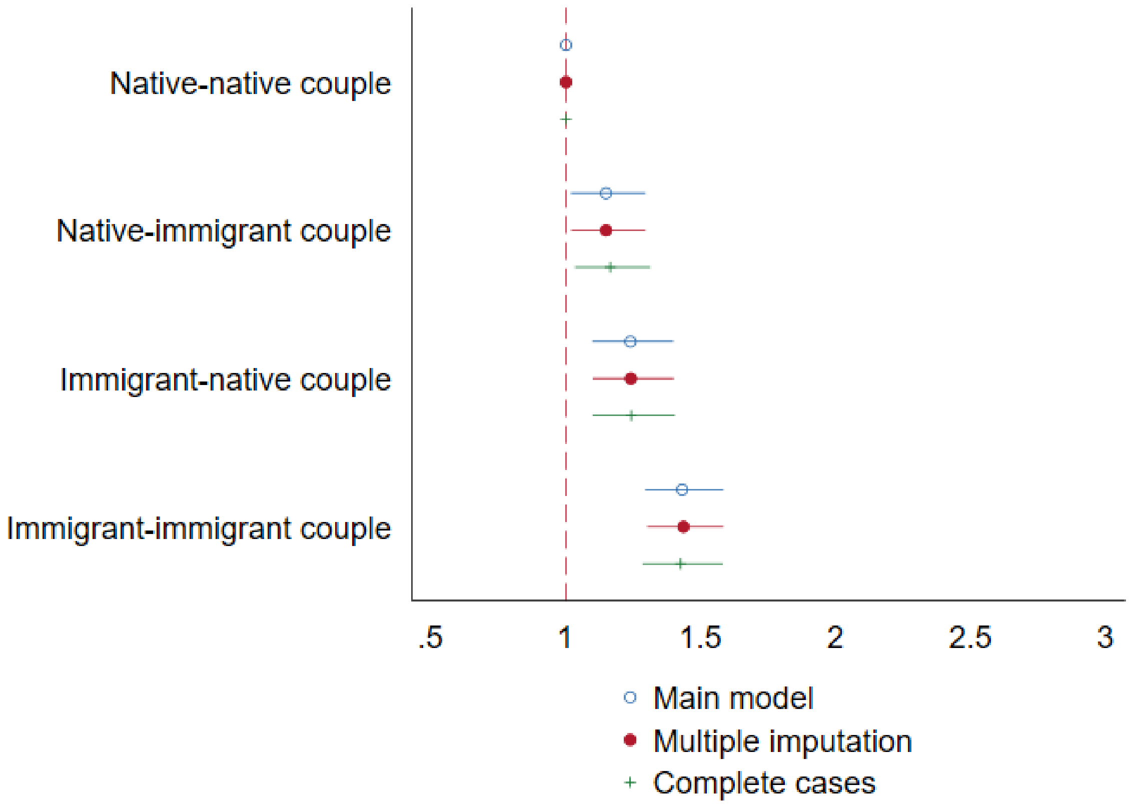

For peer review only

Supplementary figure 2: Hazard Ratios for the risk of dying from COVID-19, other causes of death during the pandemic, disaggregating native-immigrant couples by partner origin. Model includes full adjustment (reference group: native-native couples) (N=3,229,772)

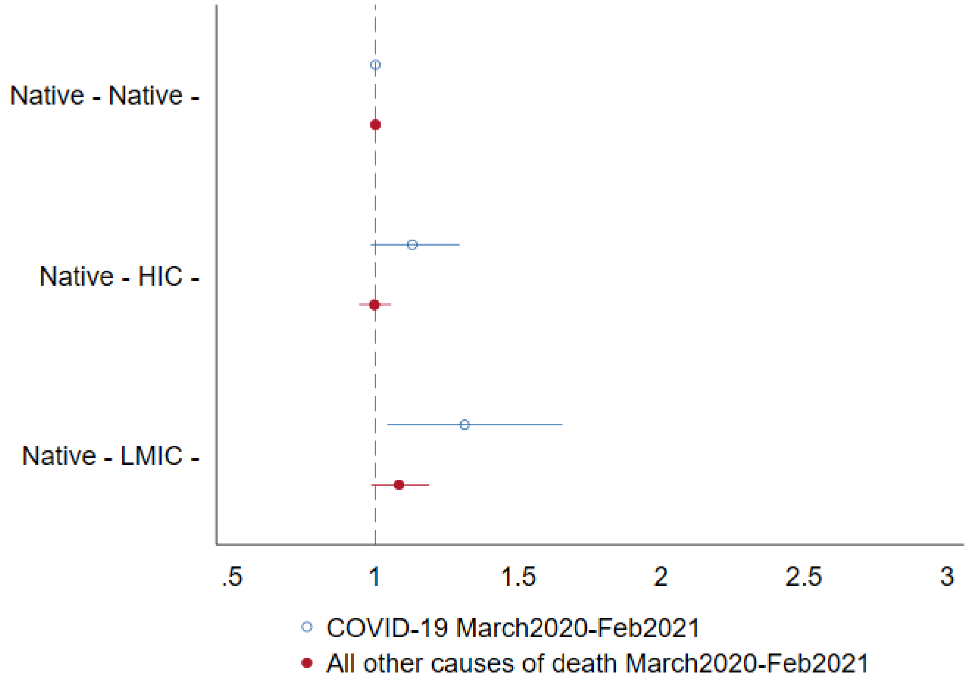

review only

Supplementary figure 3: Hazard Ratios for the risk of dying from COVID-19 by couple dyads excluding second generation immigrants. Model includes full adjustment (reference group: native-native couples) (N= 3,593,764)

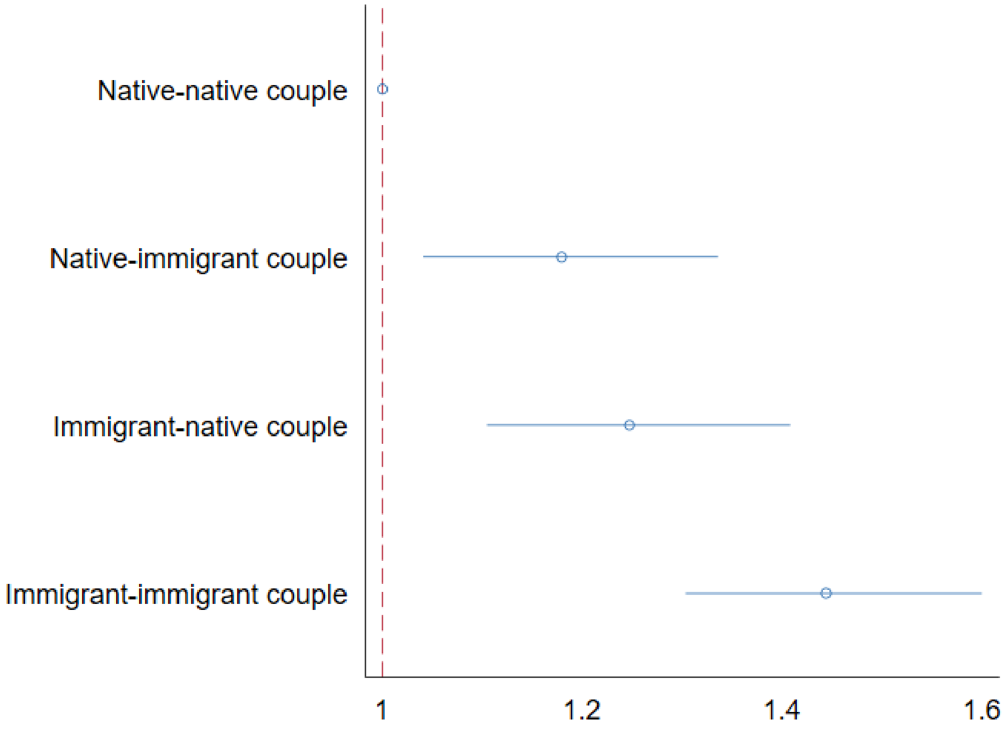

For peer review only

Supplementary figure 4: Hazard Ratios for (A) adjusted for age and sex only and (B) further adjusted associations between immigrant-native couple type (reference group: native-native couples), COVID-19 and all-cause mortality in 2020 in Stockholm, Sweden.

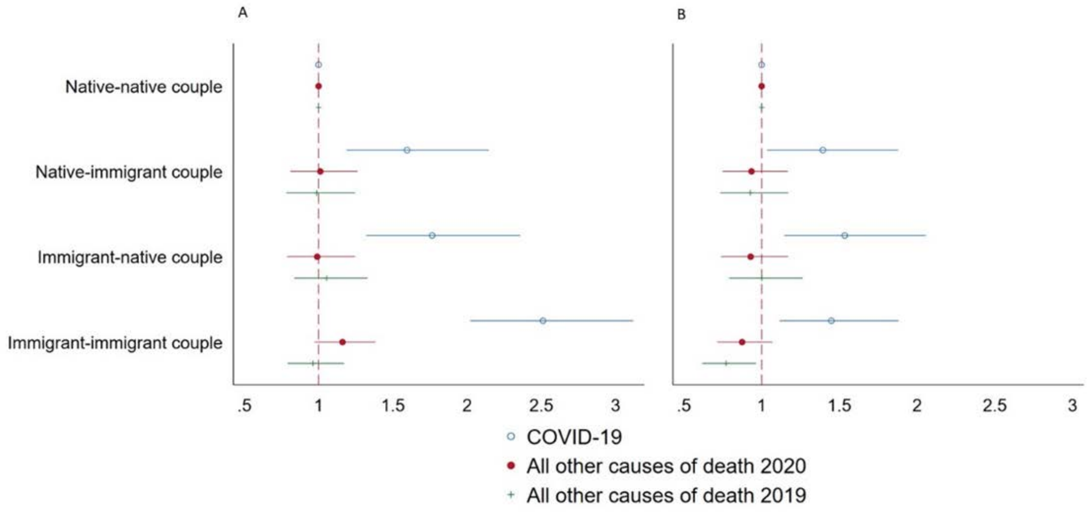

Note: This figure is based on data on all COVID-19 deaths during the period from March 12, 2020 to May 7, 2020 (149,622 person-years of observation, and 1,587 deaths).

For peer review only

STROBE Statement—checklist of items that should be included in reports of observational studies

	Item No	Recommendation
**Title and abstract**	1	(*a*) Indicate the study's design with a commonly used term in the title or the abstract **Yes**
		(*b*) Provide in the abstract an informative and balanced summary of what was done and what was found **Yes**
**Introduction**		
Background/rationale	2	Explain the scientific background and rationale for the investigation being reported **Page 5**
Objectives	3	State specific objectives, including any prespecified hypotheses **Page 5-6**
**Methods**		
Study design	4	Present key elements of study design early in the paper **Pages 6**
Setting	5	Describe the setting, locations, and relevant dates, including periods of recruitment, exposure, follow-up, and data collection **Page 6**
Participants	6	*Cohort study*—Give the eligibility criteria, and the sources and methods of selection of participants. Describe methods of follow-up **Pages 6 and page 8** *Case-control study*—Give the eligibility criteria, and the sources and methods of case ascertainment and control selection. Give the rationale for the choice of cases and controls *Cross-sectional study*—Give the eligibility criteria, and the sources and methods of selection of participants
		(*b*) *Cohort study*—For matched studies, give matching criteria and number of exposed and unexposed *Case-control study*—For matched studies, give matching criteria and the number of controls per case
Variables	7	Clearly define all outcomes, exposures, predictors, potential confounders, and effect modifiers. Give diagnostic criteria, if applicable **Pages 6-7**
Data sources/ measurement	8*	For each variable of interest, give sources of data and details of methods of assessment (measurement). Describe comparability of assessment methods if there is more than one group **Pages 6-7**
Bias	9	Describe any efforts to address potential sources of bias **Pages 8 and 9**
Study size	10	Explain how the study size was arrived at **Page 6 and figure 1**
Quantitative variables	11	Explain how quantitative variables were handled in the analyses. If applicable, describe which groupings were chosen and why **Pages 7-8**
Statistical methods	12	(*a*) Describe all statistical methods, including those used to control for confounding

For peer review only

**Pages 8-9**
(*b*) Describe any methods used to examine subgroups and interactions<br>**Pages 8-9**
(*c*) Explain how missing data were addressed<br>**Figure 1, pages 8**
(*d*) *Cohort study*—If applicable, explain how loss to follow-up was addressed<br>**Page 8**<br>*Case-control study*—If applicable, explain how matching of cases and controls was addressed<br>*Cross-sectional study*—If applicable, describe analytical methods taking account of sampling strategy
(*e*) Describe any sensitivity analyses

Continued on next page

For peer review only

For peer review only - http://bmjopen.bmj.com/site/about/guidelines.xhtml

**Results**		
Participants	13*	Report numbers of individuals at each stage of study—eg numbers potentially eligible, examined for eligibility, confirmed eligible, included in the study, completing follow-up, and analysed<br>**Not applicable**
		(b) Give reasons for non-participation at each stage<br>**Not applicable**
		(c) Consider use of a flow diagram<br>**Figure 1**
Descriptive data	14*	Give characteristics of study participants (eg demographic, clinical, social) and information on exposures and potential confounders<br>**Table 1, page 8, lines 218-225**
		(b) Indicate number of participants with missing data for each variable of interest<br>**Figure 1 and table 1**
		(c) *Cohort study*—Summarise follow-up time (eg, average and total amount)<br>**Page 8, lines 218-225, table 1**
Outcome data	15*	*Cohort study*—Report numbers of outcome events or summary measures over time<br>**page 8, lines 218-220, table 1**
		Case-control study—Report numbers in each exposure category, or summary measures of exposure
		Cross-sectional study—Report numbers of outcome events or summary measures
Main results	16	Give unadjusted estimates and, if applicable, confounder-adjusted estimates and their precision (eg, 95% confidence interval). Make clear which confounders were adjusted for and why they were included<br>**Figures 2 and 3**
		(*b*) Report category boundaries when continuous variables were categorized<br>**Table 1**
		(*c*) If relevant, consider translating estimates of relative risk into absolute risk for a meaningful time period
Other analyses	17	Report other analyses done—eg analyses of subgroups and interactions, and sensitivity analyses<br>**Page 7, lines 206-211 and page 11, lines 303-306**
**Discussion**		
Key results	18	Summarise key results with reference to study objectives<br>**Page 9-10, lines 254-265**
Limitations	19	Discuss limitations of the study, taking into account sources of potential bias or imprecision. Discuss both direction and magnitude of any potential bias<br>**Pages 11-12 lines 319-332**
Interpretation	20	Give a cautious overall interpretation of results considering objectives, limitations, multiplicity of analyses, results from similar studies, and other relevant evidence<br>**Pages 10-11, lines 267-308**
Generalisability	21	Discuss the generalisability (external validity) of the study results
**Other information**		
Funding	22	Give the source of funding and the role of the funders for the present study and, if applicable, for the original study on which the present article is based<br>**Yes**

For peer review only - http://bmjopen.bmj.com/site/about/guidelines.xhtml
For peer review only

*Give information separately for cases and controls in case-control studies and, if applicable, for exposed and unexposed groups in cohort and cross-sectional studies.

Note: An Explanation and Elaboration article discusses each checklist item and gives methodological background and published examples of transparent reporting. The STROBE checklist is best used in conjunction with this article (freely available on the Web sites of PLoS Medicine at http://www.plosmedicine.org/, Annals of Internal Medicine at http://www.annals.org/, and Epidemiology at http://www.epidem.com/). Information on the STROBE Initiative is available at www.strobe-statement.org.

For peer review only
